# Safe and effective delivery of supplemental iron to healthy adults: a two-phase, randomized, double-blind trial – the safe iron study

**DOI:** 10.3389/fnut.2023.1230061

**Published:** 2023-10-11

**Authors:** Erin D. Lewis, Edwin F. Ortega, Maria Carlota Dao, Kathryn Barger, Joel B. Mason, John M. Leong, Marcia S. Osburne, Loranne Magoun, Felix J. Nepveux V, Athar H. Chishti, Christopher Schwake, Anh Quynh, Cheryl H. Gilhooly, Gayle Petty, Weimin Guo, Gregory Matuszek, Dora Pereira, Manju Reddy, Jifan Wang, Dayong Wu, Simin N. Meydani, Gerald F. Combs

**Affiliations:** ^1^Jean Mayer USDA Human Nutrition Research Center on Aging, Tufts University, Boston, MA, United States; ^2^Department of Molecular Biology and Microbiology, Tufts University, Boston, MA, United States; ^3^Department of Developmental, Molecular and Chemical Biology, Tufts University, Boston, MA, United States; ^4^Department of Pathology, University of Cambridge, Cambridge, United Kingdom; ^5^Department of Food Science and Human Nutrition, Iowa State University, Ames, IA, United States

**Keywords:** iron, ferrous sulfate, malarial infectivity, bacterial proliferation, gut inflammation, IHAT, fungal iron

## Abstract

**Introduction:**

The safety of novel forms of iron in healthy, iron-replete adults as might occur if used in population-based iron supplementation programs was examined. We tested the hypotheses that supplementation with nanoparticulate iron hydroxide adipate tartrate (IHAT), an iron-enriched *Aspergillus oryzae* product (ASP), or ferrous sulphate heptahydrate (FS) are safe as indicated by erythrocyte susceptibility to malarial infection, bacterial proliferation, and gut inflammation. Responses to FS administered daily or weekly, and with or without other micronutrients were compared.

**Methods:**

Two phases of randomized, double-blinded trials were conducted in Boston, MA. Phase I randomized 160 volunteers to six treatments: placebo, IHAT, ASP, FS, and FS plus a micronutrient powder (MNP) administrated daily at 60 mg Fe/day; and FS administered as a single weekly dose of 420 mg Fe. Phase II randomized 86 volunteers to IHAT, ASP, or FS administered at 120 mg Fe/day. Completing these phases were 151 and 77 participants, respectively. The study was powered to detect effects on primary endpoints: susceptibility of participant erythrocytes to infection by *Plasmodium falciparum*, the proliferation potential of selected pathogenic bacteria in sera, and markers of gut inflammation. Secondary endpoints for which the study was not powered included indicators of iron status and gastrointestinal symptoms.

**Results:**

Supplementation with any form of iron did not affect any primary endpoint. Regarding secondary endpoints, in Phase I participants taking IHAT more frequently reported abdominal pain (27%, *p* = 0.008) than other iron forms; those taking the weekly FS dose more frequently reported nausea (20%, *p* = 0.009) than the other forms and modes of administration. In phase II, no such differences were observed.

**Discussion:**

With respect to the primary endpoints, few differences were found when comparing these forms of iron, indicating that 28 days of 60 or 120 mg/day of IHAT, ASP, or FS may be safe for healthy, iron-replete adults. With respect to other endpoints, subjects receiving IHAT more frequently reported abdominal pain and nausea, suggesting the need for further study.

**Clinical Trial Registration:**

ClinicalTrials.gov, NCT03212677; registered: 11 July 2017.

## 1. Introduction

Iron-deficiency affects more than a billion people worldwide, mostly children and female adults in resource-poor countries comprising a persistent global disease burden. Addressing iron deficiency through population-based iron supplementation programs has been frustrated by serious side effects of inorganic forms of iron which, due to low enteric absorption, must be given in relatively high levels (1). Those effects include gut inflammation (2–5), changes in the gut microbiome (4, 5), bloody diarrhea (2, 5–7); among iron-replete children in malaria-endemic areas increased serious morbidity has been reported (8–15). These effects are thought to involve stress on the gut by unabsorbed iron, which can be pro-oxidative and pro-inflammatory, favoring the proliferation of pathogenic enteric bacteria (14),and contributing to inflammatory responses (2, 5, 16, 17) that lead to down-regulation of iron absorption (18). Non-transferrin-bound iron (NTBI) formed from high supplements of rapidly absorbed forms of iron (e.g., ferrous sulphate, FS) has been proposed to increase malaria infection severity (19, 20) by increasing capillary sequestration of infected erythrocytes (21). Iron-replete individuals with inflammation may, thus, have increased risk of adverse effects of supplemental iron, particularly in malaria-endemic regions. Adverse effects associated with currently available forms of iron in addressing prevalent iron deficiency have effectively halted population-based iron supplementation in malaria-endemic areas.

The present study was conducted to inform the development of modalities of providing bioavailable iron with minimal adverse effects on iron-replete individuals. Three forms of iron were used: ferrous sulphate heptahydrate (FeSO_4_·7H_2_O, FS), nanoparticulate iron hydroxide adipate tartrate (IHAT), and an iron-enriched *Aspergillus oryzae* product (ASP). The novel forms of iron have been reported to have apparent bioavailability’s less than FS, as assessed by different methods (22–25). The study sought to determine whether IHAT and ASP produce fewer adverse effects than FS in iron-replete participants. The primary outcomes were: infectivity of malarial parasites (*Plasmodium falciparum*) on host erythrocytes (assessed *ex vivo*), proliferation of selected bacterial species in host plasma (assessed *ex vivo*), gut inflammation (assessed *in vivo*). Effects on iron status were also assessed. The study was conducted in two phases, each comprised of a clinical intervention trials described previously (26, 27).

## 2. Participants and methods

The Safe Iron Study design has been described previously (26). Briefly, it was a randomized, double-blind, placebo-controlled, two-phase study with iron supplementation in adults who were iron-replete. For both phases, each arm was comprised of an intervention period of 4 weeks, after which time the outcome parameters were compared between the baseline (wk 0) and post-intervention (wk 4) times.

Volunteers were recruited from the greater Boston area using advertisements in print and electronic media, flyers posted in public places, and mailings to participants in previous studies at this Center. Those who responded and gave consent were pre-screened by telephone; eligible, pre-screened individuals were invited to the Center for screening. Interested adults were asked to sign a screening consent form (28). The screening process was conducted by an experienced research study nurse. It consisted of taking a blood sample (7 ml), reviewing participant medical history and general health (including gastrointestinal health history), administering a gut irritation questionnaire, and inquiring about the use of medications and nutritional supplements including iron. Volunteers were eligible on the basis of the criteria previously described (26). Briefly, those included: *Inclusion* - apparently healthy males and post-menopausal females (no menses for ≥1 year); 50–80 years of age; BMI 18–35 kg/m^2^; typical bowel pattern of at least one stool every other day; willing to comply with study procedures; *Exclusion* - taking an iron supplement; any major illness or condition that may interfere with study outcomes at the discretion of the study physician (JBM). Volunteers deemed eligible were invited to enroll in the study at which time each was asked to complete a questionnaire recording age, body weight, height, educational level, and self-identified sex and race.

Each was free to withdraw at any time by writing, calling, or emailing the study PI (GFC). Each could be terminated if they no longer met any of the study eligibility, failed to comply with study requirements, or had adverse reaction that were considered severe in the judgment of the study physician and the PI. Each was offered a modest honorarium for participating. If a participant failed to complete the study, the honorarium was prorated accordingly, and data or specimens collected before withdrawal were used in the study.

The study protocol was approved by the Institutional Review Board of Tufts Medical Center and Tufts University Health Sciences Campus (Phase I IRB #12455, Phase II IRB#13341) which found that, because it presented minimal risk for participants, safety monitoring could be conducted by the PI (GFC) and study Physician (JBM). Nevertheless, we impaneled an external advisory board to review de-identified data provided to them periodically and assess both harms and benefits.

### 2.1. Intervention agents

The intervention agents included a placebo (Melojel® corn starch) and three forms of iron: FeSO_4_·7H_2_O (FS), IHAT, and ASP. Reagent grade FS was used. In Phase I, one arm also included a multiple micronutrient powder [MNP; MixMe™ Vitamin and Mineral Powder (DSM Nutritional Products, Geneva, Switzerland) (29)] the contents of which are based on recommendations by UNICEF/WHO/WFP for one RDA of 15 vitamins and minerals.

IHAT, developed by the Medical Research Council Elsie Widdowson Laboratory, Cambridge, UK, is composed of three GRAS substances: iron hydroxide, tartaric acid and adipic acid. It is a nano-particulate iron supplement, recently approved by the European Commission as a novel food (2022/1373; Aug. 5, 2022). IHAT is a tartrate-modified, nano-dispersed Fe(III) oxo-hydroxide with similar functional properties and small primary particle size (~2 nm) to the iron form found in the ferritin core (i.e., ferrihydrite) (30), and has been designed to have a benign side-effect profile by withholding any unabsorbed iron from redox activity and the gut flora (22, 30–33). Insoluble in the gut lumen (31, 32), IHAT appears poorly utilized by enteric bacteria (22); thus, it may be less irritating to the gut and less pro-inflammatory than FS. IHAT-iron appears to enter the metabolic iron pool more slowly than Fe-iron (33), suggesting a lesser post-absorptive surge in pro-oxidative NTBI. In female adults who were iron-deficient, the bioavailability of IHAT 75% that of FS (33). The European Food Safety Authority found the no observed adverse effect level of IHAT to be 231 mg (77 mg Fe) per kg body weight per day (34).

ASP (Aspiron™ Natural Koji Iron), a dried, iron-enriched Koji biomass containing 8–10% iron, was developed by Cura Global Health Inc. (Ames, IA). Koji is a GRAS constituent of food considered safe by the FAO/WHO Committee on Food Additives and (23, 24). Its bioavailability for the rat is of 60% of FS (25). Studies with female adults who were healthy and non-anemic with marginal iron stores (serum ferritin <40 μg/L) showed the absorption of ASP-Fe to be comparable to that of FS-Fe, but slower to appear in the plasma where it persisted longer, produced less NTBI, and produced fewer side effects (27, 35, 36).

Each of these agents was approved for human use. Each was encapsulated in opaque, double-zero gelatin capsules identical in size, color, and weight. This was done by the Richardson Centre for Functional Foods and Nutraceuticals, University of Manitoba (Winnipeg, Manitoba, Canada), which is licensed as a Health Canada Natural Health Product Site with the ability to produce good manufacturing practices and Hazard Analysis and Critical Control Points certified products.

### 2.2. Randomization

**Figure 1:**
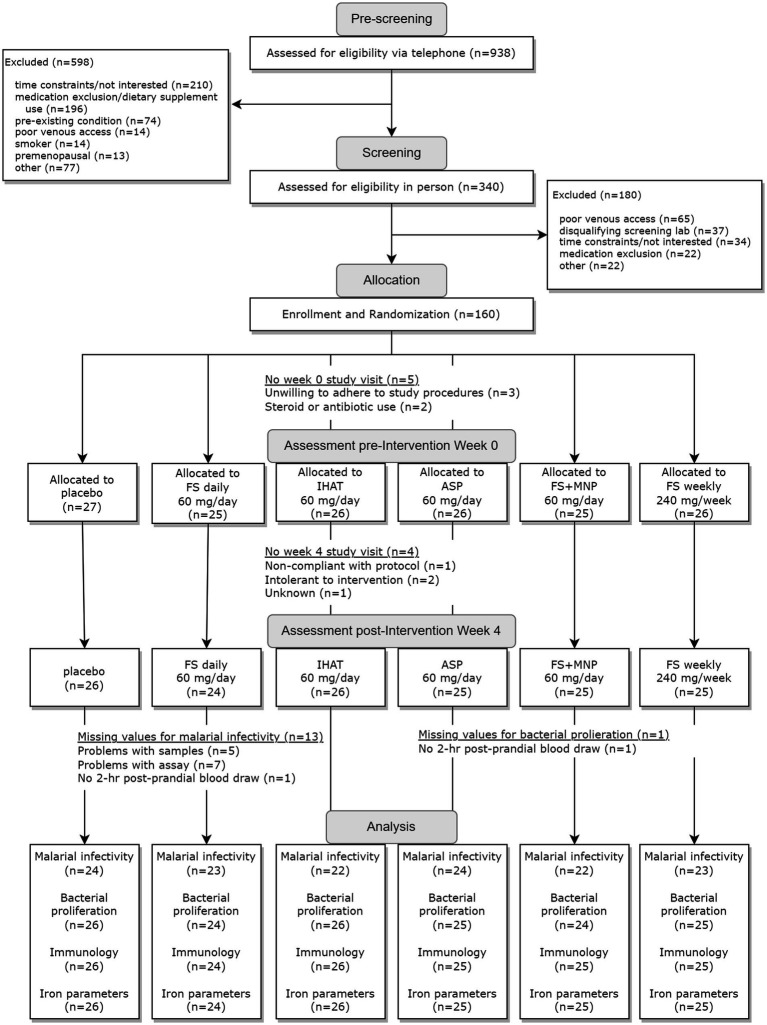
Flow diagram for study Phase I.

**Figure 2:**
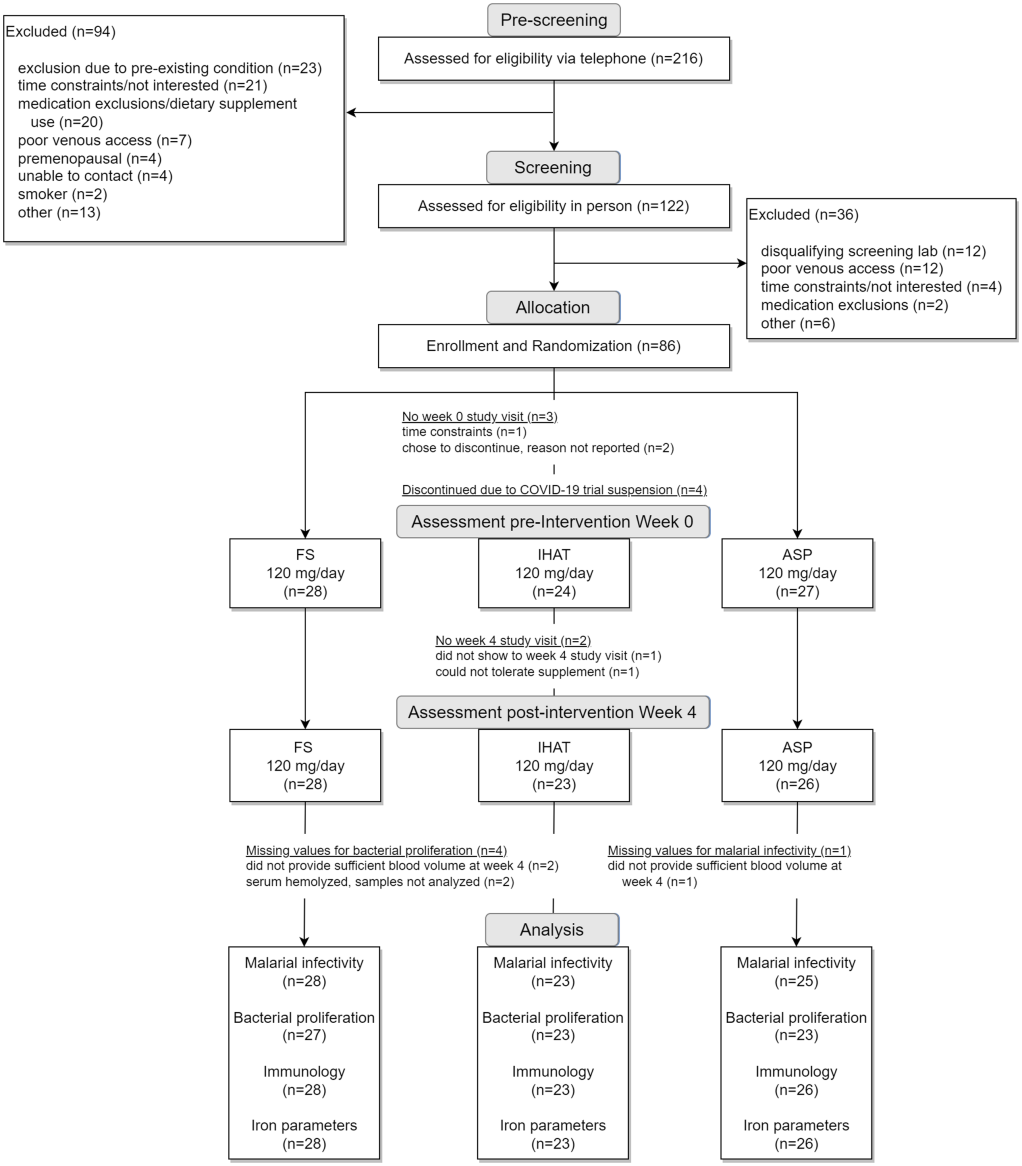
Flow diagram for study Phase II.

The two phases of this study were conducted sequentially, i.e., Phase I (Figure 1) was completed in its entirety prior to the initiation of Phase II (Figure 2). For each phase of the study, a randomization scheme for entering participants was generated using SIMD-oriented Fast Mersenne Twister algorithm (v. 1.5.1)-based randomization functions in the *StatsBase.jl* Julia package,^1^ stored, and known only to the data manager who held the unblinded key under lock and key data collection for the entire study. For Phase I, 160 participants were randomly assigned to one of the six treatment arms at the time they were enrolled in the study (Figure 1): placebo; FS daily (60 mg iron/d); FS weekly (420 mg iron/wk); FS + MNP (60 mg iron/d); IHAT (60 mg iron/d); and ASP (60 mg iron/d). The dose of 60 mg iron /d was based on the WHO recommendation for daily supplementation for non-anemic, pregnant females (30–60 mg iron/d) (37). A separate treatment consisting of weekly supplementation of 420 mg iron in four capsules taken once weekly; this dose regimen has been recommended as feasible, cost-effective in population-base programs (38–40), and may produce fewer gastrointestinal complaints than daily FS doses. In addition, a treatment involving the co-administration of FS and MNP was included to examine the safety of this common practice, as components of MNP, particularly ascorbic acid, have been shown to promote the enteric absorption of ferrous iron (41). The corn starch placebo provided a control for prospective changes over time, effects of daily ingesting capsules, and any time-related and/or and seasonal effects. For Phase II, 86 participants were randomly assigned to one of three treatment arms (Figure 2): IHAT (120 mg iron/d); ASP (120 mg iron/d); and FS (120 mg iron/d). All groups received four physically similar capsules to ensure blinding of the treatments. The dose of 120 mg Fe/day was based on the report of the Food and Nutrition Board (42), which suggest low adverse effects levels (constipation, diarrhea, black stools, gut irritation) of FS at that dose. Because this phase comprised direct comparisons between the three sources of iron, placebo control was not included.

At enrollment, informed consent was obtained and instructions for stool sample collection were reviewed. The participant was then randomly assigned to a treatment group and was scheduled for a baseline, pre-intervention visit (wk 0) at which a stool sample and a fasting blood sample (7 ml) were collected, and the participant was then given a standard continental breakfast (English muffin or bagel, spreads, coffee, juice, fruit excluding orange products, and teas). Immediately following the breakfast, the participant was given the assigned blinded intervention agent for the baseline (wk 0) dose. Instructions for the intervention were reviewed, and the participant was sent home with a pre-loaded calendar pack containing 90 capsules (three to be taken each day for 30 days). For the weekly FS group, treatment concealment was deemed impractical and therefore was not used; each participant received 16 capsules (four to be taken once weekly for 4 weeks). Each instructed to take all their scheduled capsules within a few minutes, was also given a compliance calendar to record capsule intake and was instructed to return the calendar with any unused capsules at the final study visit. Adherence to supplement protocol was assessed by counting the number of capsules remaining in the returned planner. Two hours after the breakfast, a second, post-prandial (PP) blood sample (7 ml) was drawn for use in bacterial proliferation assays.

### 2.3. Primary endpoints

The study was powered to detect treatment effects on the following endpoints:

#### 2.3.1. *Ex vivo* assessment of malarial infectivity

Red blood cells (RBCs) were prepared from whole blood by three washes with RPMI-1640 (4°C), with recovery by low-speed centrifugation (500 × *g* for 10 min at 4°C). Packed cells were mixed with an equal volume of a freezing solution (28% glycerol, 3% mannitol, 0.65% sodium chloride); 1 ml aliquots were flash frozen and held at −80°C. For use in the invasion assay, samples were thawed with a stepwise sodium chloride gradient (43). *Plasmodium falciparum* 3D7 parasites were cultured *in vitro* in complete malaria media (CMM) containing RPMI-1640 supplemented with 0.5% Albumax II, 25 mM HEPES, 50 mg/L Hypoxanthine, 50 mg/L Gentamicin at 37°C and maintained with a gas mixture of 5% CO_2_, 3% O_2_, balanced by N_2_ as described previously (44). Malarial viability was confirmed by microscopic examination of blood smears.

For the invasion assay, *P. falciparum* schizonts were added in a 96-well plate, each well containing 5 μl of packed participant RBCs at a starting parasitemia of 0.5–1% per well. After incubation for 24 h at 37°C, when schizont concentration reached ca. 2.5% in a total volume of 200 μl CMM, parasites were fixed using a solution of 2% paraformaldehyde and 0.2% glutaraldehyde in PBS at 4°C for 45 min. Parasitemia was quantified by flow cytometry using an LSRII instrument (BD Biosciences, San Jose, CA, USA) with staining with Hoechst 33342 dye (Mafatlal Dyes and Chemicals Ltd., Mubai, Mahrashtra, India) and a 450/65 filter to measure the signal. A total of 100,000 events were captured for each measurement. Each assay was performed in duplicate and included two kinds of controls: an uninfected sample of participant RBCs, and a sample of non-frozen blood to normalize for any differences observed in parasite infectivity between invasion assays. For each participant, the 4-week change in malarial infectivity was quantified using the logarithm of the ratio of percent parasitized erythrocytes at baseline (wk 0) and post-intervention (wk 4).

#### 2.3.2. *Ex vivo* assessment of bacterial proliferation potential

The potential for participant plasma to facilitate the proliferation of pathogenic bacteria was assessed *ex vivo* using five bacterial strains: (a) *Staphylococcus aureus* MW2 (45), an important cause of global morbidity and mortality (46); (b) *Acinetobacter baumannii* EGA50 [AB307-0294] (47), a nosocomial pathogen (48); (c) *S. enterica*, serovar *Typhimurium* (49), which can cause life-threatening bacteremia in young African children (50) and is closely related to *S. enterica*, serovar *Typhi*, an important cause of sepsis in low- and middle-income countries (51); (d) *Klebsiella pneumoniae* MGH 78578 (52), an opportunistic pathogen (53); and (e) extraintestinal pathogenic *Escherichia coli* ST131 [ExPEC] (54), an important cause of bacteremia (55).

Iron-depleted bacterial growth media was generated by adding 25 g Chelex 100 resin (BioRad #1421253) to 500 ml of LB media in a sterile container with stirring for 1 h at RT. The resin was then removed by filtration through a 0.22 μm filter. Aliquots of participant serum were heated at 55°C for 30 min to inactivate complement, and then held at −80°C until use. Each bacterial strain was grown on LB agar plates overnight at 37°C; they were harvested by scraping the plate and resuspended in 3 ml Chelex-treated LB. Bacterial suspensions were diluted in Chelex-treated LB to a final Optical density at 600 nm (OD_600_) of 0.5. Growth assays were performed in 96-well microtiter plates. All five bacteria were tested for growth on the same plate in sera from a single participant (1.25 ml aliquots of each pre- (wk 0) and post-supplementation (wk 4) serum were thawed at RT for 1 h prior to use). Chelex-treated LB (45 μl) was inoculated with 5 μl of each 0.5 OD_600_ bacterial suspension, and then mixed with 50 μl participant serum in triplicate wells each of which were then covered with 50 μl light mineral oil to minimize evaporation and cross contamination. Covered plates were a continuously slow shaking plate reader at 37°C. OD_600_ was determined then and subsequently at 20 min intervals for 18.5 h. Three controls were included on each plate: growth in iron-replete LB to confirm bacterial viability and growth consistency; participant serum alone with Chelex-treated LB to confirm serum sterility; and wells containing either LB or Chelex-treated LB to normalize OD readings and assess well-to-well cross contamination. Contamination was rare and never resulted in data elimination.

Bacterial proliferation data were modeled using polynomial growth curves similar to those previously reported (56). Eighteen-hour growth curves were fit from the mean OD of three replicates measured every 20 min, with OD expressed in natural logarithm. Fourth degree polynomials were fit using ordinary least squares. Three growth parameters were calculated from the estimated curves: maximum OD (max OD): the normalized maximum optical density reached after the exponential growth period, peak growth rate index (the normalized rate during the exponential growth period, i.e., the steepest slope of the growth curve in the log OD vs. time plot), and time to peak growth (in hours). Max OD and peak growth rate are expressed as indices to indicate the values were normalized. Control values on each plate were used to normalize growth parameters to account for day-to-day variation and utilization of different plate readers, except for time to peak growth due to a high proportion of zero values.

#### 2.3.3. Markers of gut inflammation

Gut inflammation was assessed using multiple fecal biomarkers of intestinal inflammation. Participant stool samples were collected in plastic bags, cooled with frozen gel packs, and homogenized and extracted within 24 h using phosphate-buffered saline containing 1 mM phenylmethylsulfonyl fluoride, 1% bovine serum albumin, and 0.05% Tween-20. Extracts were held at −80°C until analysis. Sandwich ELISA kits were used to determined fecal concentrations of calprotectin (R&D Systems, Minneapolis, MN), myeloperoxidase (R&D Systems, Minneapolis, MN) and *α*-1-antitrypsin (Cat. #: DY1268, R&D Systems, Minneapolis, MN). The fecal immunochemical test (FIT) for occult blood in stool samples (57) was performed by the Hematology Laboratory, Tufts Medical Center.

### 2.4. Secondary endpoints

Also addressed were the following secondary endpoints for which the study was not powered:

#### 2.4.1. Assessment of general health status and dietary patterns

**Figure 3:**
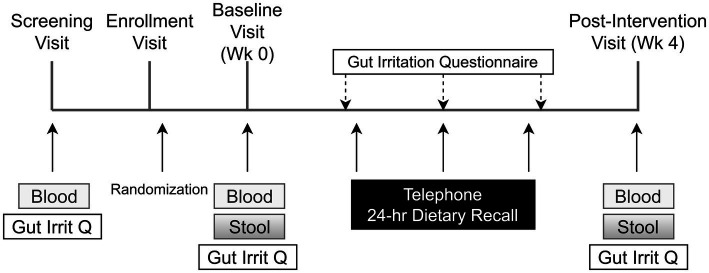
Schedule of subject activities in both phases of the study. The title of each figure is shown above. Figure legends are not applicable.

In each phase, the health of each participant was monitored weekly. The study physician (JBM), a practicing gastroenterologist, was available for support. Each participant was also given a 24 h dietary recall questionnaire administered weekly by telephone (Figure 3). Recalls were analyzed by the multiple-pass 24-h recall method, using the Nutrition Data System for Research software v. 2016 and v. 2020 (Nutrition Coordinating Center, University of Minnesota, Minneapolis, MN).

#### 2.4.2. Assessment of iron status

Hemoglobin and hematocrit were determined in whole blood as part of a 20-parameter hematology profile determined by cytochemistry, impedance, and analysis of cellular structure by light absorbance using a hematology analyzer (Pentra 60c + Hematology Analyzer, HORIBA ABX SAS, HORIBA Instruments Incorporated, Albany, NY). Serum iron was measured by an endpoint colorimetric procedure (58) using a clinical chemistry analyzer (AU480 Clinical Chemistry Analyzer, Beckman Coulter, Inc., Brea, CA). Unsaturated iron binding capacity in serum was measured by a colorimetric procedure using a clinical chemistry analyzer (AU480 Clinical Chemistry Analyzer, Beckman Coulter, Inc., Brea, CA). Average intra- and inter-assay CVs were less than 4.0 and 6.0%, respectively. Total iron binding capacity (TIBC) was determined from the total serum iron and unsaturated iron binding capacity measured using a clinical chemistry analyzer (AU480 Clinical Chemistry Analyzer, Beckman Coulter, Inc., Brea, CA). Transferrin was determined in serum by an immunoturbidimetric assay using a clinical chemistry analyzer (AU480 Clinical Chemistry Analyzer, Beckman Coulter, Inc., Brea, CA); transferrin saturation was calculated from the relationship of serum iron to total iron-binding capacity. Average intra- and inter- assay CVs were <2.5 and <3.0%, respectively. Ferritin was measured in serum by solid-phase, two-site chemiluminescent immunometric assays using the IMMULITE 2000 (Siemens Healthcare Diagnostics, Los Angeles, CA) according to Babson (59). The average intra- and inter-assay CVs were less than 5 and 6%, respectively.

#### 2.4.3. Assessment of gastrointestinal symptoms

A questionnaire designed and validated to address symptoms of gastrointestinal irritation after FS supplementation (60) was administered after the initial fasting blood draw and again weekly by telephone.

### 2.5. Data management

Data were managed using a REDCap (61) database and handled in compliance with HIPAA and 21CFR11. Participant identifiers were recorded in separate electronic case reports and were maintained separately from research data. Each participant’s identity and records of telephone pre-screening, final screening, and study data were kept in locked files or eCRF, with only the PI (GFC), Study Physician (JBM), and Study Coordinators having access to participant identifying information if needed. Otherwise, only de-identified data were made available to study co-investigators. Data entered into the database underwent external validation checks; meta-data files and data dictionaries were used to provide information necessary for proper use and understanding of the data files. Laboratory specimens were identified by unique study codes; the master list linking the identities of participants and specimens was kept in a locked file and on a separate, password-protected database on a secure server. REDCap was used for edit resolution; trouble shooting data entry problems; an audit trail of database editing; performing range checks for cleaning, daily backup, cleaning of transitional databases; and, ultimately, transferring master databases for statistical analysis. At key points in data acquisition, clean-up and analysis, an additional off-site copy of the data was stored using the LabArchives electronic laboratory notebook software (LabArchives LLC, San Marcos, CA, USA). Access to the study database was restricted until the study was completed and unblinded. Protected participant health information was automatically de-identified for all viewing and data exports.

### 2.6. Statistical methods

The sample sizes used in both phases were based on power calculations for each primary outcome as previously described (26). For malarial infectivity, a standard deviation (SD) of 0.202 was estimated from published studies with *P. falciparum* infectivity (62). Based on an independent two-sample t-test with 0.05 type I error, it was determined that 25 participants per group would yield 90% power to detect a 20% reduction in parasite infectivity. For bacterial proliferation, between-participant estimates of SD for doubling time during the exponential phase of growth were estimated for several species from published data (59). This indicated that, with 25 participants per treatment group and 0.01 type I error (adjusted for planned analyses with five species), power was >0.99 for a minimal detectable difference in doubling time of 1 h. Mean fecal calprotectin was reported previously to be 1.9 μg/g in participants with minimal inflammation (irritable bowel syndrome) and 27.6 μg/g in participants with mild inflammation (collagenous colitis) with a SD of 1.3 in log concentration (63). A sample size of 25 participants per group was estimated to allow the detection of a 3.5-fold change in fecal calprotectin values with 90% power at a type I error level of 0.05.

An intention-to-treat analysis was performed with all participants according to their randomization assignment. Analysis excluding participants with low supplement adherence was also performed, however did not substantially affect results; thus, the results from the intention-to-treat approach are presented. Participant characteristics and dietary intake were summarized for each intervention group using mean, median, or frequency, along with SD, range, or interquartile range, as appropriate. Statistical testing was structured to test the study hypotheses and objectives in a series of planned pair-wise comparisons. The formal hypotheses of the study (26) were tested by evaluating the following outcomes: metabolic responses as assessed by biomarkers of iron status; the susceptibility of participant RBCs to infection by *P. falciparum* as assessed *ex vivo*; the ability of participant plasma to facilitate proliferation of selected species of pathogenic bacteria as assessed *ex vivo*; and inflammatory responses as assessed by fecal biomarkers of inflammation. Treatment effects were evaluated by the following sets of pairwise comparisons:

- Phase I (six treatments each providing 60 mg Fe/day):
  - New iron forms: IHAT vs. FS; ASP vs. FS; FS vs. placebo; IHAT vs. placebo; and ASP vs. placebo
  - Modes of administration: FS daily vs. FS + MNP daily; and FS daily vs. FS weekly
- Phase II (three treatments each providing 120 mg Fe/day):
  - New iron forms: IHAT vs. FS; ASP vs. FS; IHAT vs. ASP

An alpha level was designated for each set of comparisons to achieve a familywise error rate of 0.05 for each outcome. Models were fit using the lmer4 package in R v. 4.2 and *p* values from pairwise comparisons were adjusted based on Tukey’s HSD method applied in the emmeans package v. 1.7.4-1 (64). Four-week changes were assessed using linear models adjusted for age, sex, and BMI. A participant-specific random effect was added to the models for outcomes that were assessed both at fasting and 2-h PP. Additionally, noninferiority tests were performed for the primary outcomes in Phase I. These tests were not prespecified but were added to the analysis to characterize comparisons of the supplemental iron forms to FS daily, given the interpretation of these comparisons as one-sided equivalence hypotheses. A conservative noninferiority margin was set at 50% of the 4 wk. change between placebo and FS daily. Data were evaluated graphically to assess modeling assumptions including outlier detection, normality, and linearity with covariates. Log transformations were used when appropriate to satisfy normality assumptions.

## 3. Results

### 3.1. Results of phase I

#### 3.1.1. Participants

The Phase I recruitment scheme is shown in Figure 1. This produced a cohort of 160 participants comprised of mostly college-educated females and males aged 50–80 yrs. (Table 1), recruited between June 2016 and June 2019. During the study, participants consumed ca. 1940 kcal/day, including ca. 78 g/day of protein more than half of which was from foods of animal origin. The estimated dietary iron intakes of participants were 12–15 mg/day (Table 1). Of the 151 participants that completed the study, 142 reported the number of returned capsules. Adherence is reported as the number of participants with an intake of at least 80% of the total provided supplements based on returned capsules. All participants on daily supplementation regimens reported taking >80% of the allocated capsules during the 4-week study, whereas 23 of the 25 participants in the weekly supplementation group reported >80% adherence.

**Table 1:** Baseline characteristics of participants in Phase I.

Characteristic^1^	Treatment group					
	Placebo (*n* = 27)	FS daily 60 mg Fe (*n* = 25)	IHAT 60 mg Fe/d (*n* = 26)	ASP 60 mg Fe/d (*n* = 26)	FS + MNP 60 mg Fe/d (*n* = 25)	FS weekly 420 mg Fe (*n* = 26)
Age, y						
Mean (SD)	64.4 (7.1)	64.0 (5.9)	66.0 (7.4)	65.8 (7.0)	64.7 (6.9)	61.8 (7.7)
Range	50–78	55–77	50–77	53–78	51–74	50–79
Sex						
Female, %	8 (30)	11 (44)	15 (58)	10 (38)	13 (52)	15 (58)
BMI, kg/m^2^						
Mean (SD)	25.9 (4.0)	24.6 (3.6)	25.1 (3.5)	26.2 (2.8)	24.4 (3.2)	23.9 (2.9)
Range	21–34	19–34	20–35	21–32	19–30	18–32
Race (*n* = 155)						
White/Caucasian, n (%)	21 (81)	22 (92)	21 (81)	20 (77)	18 (72)	17 (65)
Other, n (%)	5 (19)	2 (8)	5 (19)	6 (23)	7 (28)	9 (35)
Education completed (*n* = 138)						
<4 y college, *n* (%)	5 (20)	4 (17)	4 (15)	3 (12)	6 (25)	2 (8)
4 y college, *n* (%)	7 (28)	7 (29)	11 (42)	11 (46)	10 (42)	15 (58)
Grad school or more, *n* (%)	13 (52)	13 (54)	11 (42)	10 (42)	8 (33)	9 (35)
Dietary intake, median (IQR^2^)						
Total energy intake, kcal/d	1910 (1,523, 2,541)	1991 (1,693, 2,180)	1812 (1717, 2098)	2014 (1,519, 2,327)	1998 (1,591, 2,315)	1924 (1,539, 2,399)
Total protein intake, g/d	77 (63, 103)	83 (63, 103)	81 (63, 95)	79 (60, 101)	80 (68, 100)	70 (54, 87)
% Animal protein	62 (54, 70)	59 (39, 68)	63 (45, 69)	57 (47, 67)	60 (52, 68)	57 (48, 64)
% Plant protein	38 (30, 45)	41 (32, 61)	37 (29, 55)	43 (33, 53)	40 (32, 48)	43 (36, 52)
Iron intake, mg/d	13 (11, 15)	14 (13, 20)	12 (11, 16)	15 (12, 19)	15 (10, 19)	13 (10, 17)

1 Sample sizes due to missing data are indicated for individual variables.

2 Interquartile range.

#### 3.1.2. Primary endpoints

- ***Ex vivo* malarial invasion**. No form of Fe affected the 28-day change in *ex vivo* susceptibility of participant erythrocytes to *P. falciparum* parasitemia, and that the mode of administering FS (daily *v.* weekly) had no effect on that endpoint (Table 2). None of the supplemental iron groups showed evidence of noninferiority to FS daily (Supplementary Table 2).
- ***Ex vivo* bacterial proliferation**. No form of iron affected the *ex vivo* proliferation of *E. coli, K. pneumoniae, S. aureus,* or *S. typhimurium* when assessed in participant fasting plasma (Table 3). However, when assessed in 2-h PP sera, the 4-week changes in max OD and peak growth rate index for *Acinobacter baumannii* were significantly different (*p* = 0.041 and *p* = 0.015, respectively) for participants given ASP as compared to those given FS; these data are included in the Supplementary Data (Supplementary Table 1). For peak growth rate in *E.coli* and *S. typhimurium*, a noninferiority margin was established using data from the placebo group. IHAT was shown to be noninferior to FS daily for both *E.coli* (*p* = 0.049 for noninferiority) and *S. typhimurium* (*p* = 0.005 for noninferiority). Additionally, noninferiority of ASP, FS + MNP and FS weekly were statistically significant for *S. typhimurium* (Supplementary Table 2).
- **Gut inflammation**. No form of Fe affected the 4-week changes in any markers of gut inflammation (Table 4). Moreover, none of the markers at week four in any intervention groups were significantly different than that observed in the placebo group. IHAT was shown to be noninferior to FS daily for fecal calprotectin (*p* = 0.04 for noninferiority).

**Table 2:** Phase I results: *Ex vivo* malarial invasion of participant’s erythrocytes.

Week	Treatment					
	Placebo (*n* = 24)	FS daily 60 mg Fe/d (*n* = 23)	IHAT 60 mg Fe/d (*n* = 22)	ASP 60 mg Fe/d (*n* = 24)	FS + MNP 60 mg Fe/d (*n* = 22)	FS weekly 420 mg Fe (*n* = 23)
	Parasitemia % ratio (24 h/0 h), mean [95% CI]^1^					
0	2.75 [2.30, 3.28]	2.95 [2.39, 3.65]	2.57 [2.11, 3.14]	2.20 [1.76, 2.75]	2.83 [2.30, 3.49]	2.73 [2.30, 3.25]
4	2.83 [2.42, 3.31]	3.54 [2.99, 4.19]	3.01 [2.42, 3.75]	3.08 [2.69, 3.52]	3.01 [2.36, 3.84]	2.85 [2.39, 3.40]
Δ^2^	1.03 [0.82, 1.30]	1.20 [0.97, 1.49]	1.17 [0.94, 1.45]	1.40 [1.14, 1.71]	1.06 [0.85, 1.33]	1.04 [0.80, 1.36]

ANOVA results: Pairwise comparisons (FS daily vs. placebo, IHAT vs. placebo, ASP vs. placebo, IHAT vs. FS daily, ASP vs. FS daily) and (FS daily vs. FS + MNP, FS daily vs. weekly) of Δ values showed no significant differences (Tukey HSD adjusted *p* values > 0.05) in a linear mixed-effects model adjusted for age, sex, and BMI.

1 Geometric means and 95% confidence intervals based on log-transformed data.

2 Δ = wk 4/wk 0, paired ratios.

**Table 3:** Phase I results: *Ex vivo* proliferation of bacteria in presence of participants’ plasma.

Bacterium	Growth parameter	Week	Treatment					
			Placebo (*n* = 26)	FS daily 60 mg Fe/d (*n* = 24)	IHAT 60 mg Fe/d (*n* = 26)	ASP 60 mg Fe/d (*n* = 25)	FS + MNP 60 mg Fe/d (*n* = 24)	FS weekly 420 mg Fe (*n* = 25)
			Mean [95% CI]^1^					
*Escherichia coli*	Max OD index	0	0.53 [0.48, 0.59]	0.46 [0.40, 0.52]	0.50 [0.44, 0.56]	0.49 [0.44, 0.54]	0.47 [0.41, 0.53]	0.48 [0.42, 0.54]
		4	0.46 [0.40, 0.52]	0.42 [0.37, 0.46]	0.39 [0.35, 0.43]	0.39 [0.35, 0.43]	0.43 [0.37, 0.48]	0.45 [0.39, 0.51]
		Δ^2^	−0.07 [−0.13, −0.01]	−0.04 [−0.09, 0.004]	−0.11 [−0.17, −0.05]	−0. 10 [−0.15, −0.05]	−0.04 [−0.08, −0.01]	−0.03 [−0.12, 0.06]
	Peak growth rate index^3^	0	0.60 [0.59, 0.62]	0.59 [0.57,0.61]	0.59 [0.57,0.60]	0.59 [0.57, 0.60]	0.59 [0.57, 0.61]	0.60 [0.58, 0.62]
		4	0.60 [0.58, 0.63]	0.59 [0.57,0.61]	0.58 [0.56,0.60]	0.58 [0.57, 0.60]	0.59 [0.57, 0.61]	0.60 [0.58, 0.62]
		Δ	0.001 [−0.02, 0.02]	0.002 [−0.01, 0.01]	−0.007 [−0.02,0.001]	−0.005 [−0.01, 0.003]	−0.001 [−0.008,0.007]	0.004 [−0.009,0.017]
	Time to peak growth, hour^4^	0	0.01 [−0.01, 0.03]	0.00 [0.00, 0.01]	0	0	0.001 [−0.01, 0.003]	0
		4	0.03 [−0.03, 0.09]	0	0.02 [−0.03. 0.08]	0	0	0
		Δ	0.02 [−0.02, 0.06]	−0.003 [−0.01, 0.003]	0.025 [−0.03, 0.08]	0	−0.001 [−0.003, 0.001]	0
*Acinetobacter baumannii*	Max OD index	0	0.77 [0.75, 0.80]	0.77 [0.75, 0.80]	0.77 [0.73, 0.81]	0.74 [0.71, 0.78]	0.79 [0.75, 0.82]	0.76 [0.73, 0.79]
		4	0.78 [0.75, 0.81]	0.76 [0.73, 0.79]	0.76 [0.73, 0.79]	0.76 [0.74, 0.78]	0.77 [0.73, 0.81]	0.77 [0.73, 0.81]
		Δ	0.003 [−0.02, 0.03]	−0.01 [−0.04, 0.01]	−0.01 [−0.04, 0.02]	0.02 [−0.02, 0.05]	−0.01 [−0.04, 0.02]	0.003 [−0.030, 0.036]
	Peak growth rate index	0	0.69 [0.66, 0.71]	0.68 [0.66, 0.69]	0.67 [0.65, 0.69]	0.67 [0.65, 0.69]	0.69 [0.66, 0.71]	0.68 [0.66, 0.70]
		4	0.69 [0.67, 0.71]	0.68 [0.66, 0.69]	0.67 [0.65, 0.68]	0.67 [0.65, 0.70]	0.69 [0.66, 0.71]	0.68 [0.66, 0.70]
		Δ	0.002 [−0.003, 0.006]	0.00 [−0.004, 0.004]	−0.002 [−0.006, 0.003]	0.002 [−0.003, 0.007]	0.001 [−0.003, 0.004]	0.001 [−0.005, 0.007]
	Time to peak growth, hour	0	7.22 [6.90, 7.53]	7.29 [6.96, 7.62]	7.08 [6.75, 7.41]	7.02 [6.70, 7.35]	7.07 [6.75, 7.39]	7.19 [6.77, 7.60]
		4	7.30 [6.93, 7.67]	7.29 [6.95, 7.64]	7.25 [6.96, 7.55]	7.12 [6.74, 7.50]	7.03 [6.79, 7.27]	7.35 [6.89, 7.80]
		Δ	0.09 [−0.11, 0.28]	0.01 [−0.23, 0.24]	0.18 [−0.10, 0.46]	0.10 [−0.17, 0.36]	−0.04 [−0.23,0.15]	0.16 [−0.13,0.45]
*Klebsiella pneumoniae*	Max OD index	0	0.26 [0.24,0.29]	0.26 [0.23, 0.30]	0.26 [0.23, 0.30]	0.27 [0.25, 0.30]	0.27 [0.32, 0.31]	0.26 [0.22, 0.30]
		4	0.28 [0.24, 0.32]	0.25 [0.22, 0.27]	0.25 [0.23, 0.27]	0.27 [0.25, 0.30]	0.28 [0.24,0.32]	0.24 [0.21,0.28]
		Δ	0.02 [−0.01, 0.04]	−0.02 [−0.05, 0.02]	−0.01 [−0.04, 0.01]	0.00 [−0.02, 0.02]	0.01 [−0.01, 0.02]	−0.02 [−0.04, 0.01]
	Peak growth rate index	0	0.48 [0.46, 0.49]	0.47 [0.46, 0.48]	0.47 [0.46, 0.47]	0.48 [0.47, 0.50]	0.48 [0.46, 0.50]	0.47 [0.46, 0.49]
		4	0.48 [0.47, 0.50]	0.47 [0.46, 0.48]	0.47 [0.46, 0.47]	0.49 [0.47, 0.50]	0.49 [0.47, 0.51]	0.48 [0.47, 0.49]
		Δ	0.006 [0.001, 0.010]	0.001 [−0.005, 0.007]	0.001 [−0.004, 0.006]	0.003 [−0.004. 0.009]	0.007 [−0.003, 0.018]	0.005 [−0.001, 0.011]
	Time to peak growth, hour	0	1.38 [0.60, 2.16]	1.04 [0.45, 1.62]	2.28 [1.27, 3.29]	1.50 [0.62, 2.38]	1.46 [0.61, 2.32]	1.79 [1.02, 2.55]
		4	1.14 [0.48, 1.79]	1.05 [0.35, 1.74]	1.80 [0.87, 2.74]	1.33 [0.55, 2.11]	1.04 [0.26, 1.83]	1.45 [0.65, 2.25]
		Δ	−0.25 [−1.13, 0.64]	0.013 [−0.56, 0.58]	−0.48 [−1.27, 0.32]	−0.17 [−0.74, 0.39]	−0.42 [−0.89, 0.05]	−0.34 [−0.83, 0.16]
*Staphylococcus aureus*	Max OD index	0	0.71 [0.65, 0.77]	0.77 [0.71, 0.83]	0.77 [0.71, 0.82]	0.74 [0.67, 0.80]	0.78 [0.73, 0.84]	0.77 [0.72, 0.82]
		4	0.72 [0.66, 0.78]	0.76 [0.70, 0.82]	0.77 [0.71, 0.83]	0.73 [0.67, 0.79]	0.76 [0.68, 0.83]	0.75 [0.69, 0.81]
		Δ	0.01 [−0.02, 0.05]	−0.01 [−0.04, 0.02]	0.00 [−0.03, 0.03]	−0.01 [−0.03, 0.02]	−0.03 [−0.09, 0.04]	−0.02 [−0.06, 0.03]
	Peak growth rate index	0	0.81 [0.77, 0.85]	0.81 [0.75, 0.87]	0.78 [0.73, 0.83]	0.83 [0.77, 0.89]	0.83 [0.75, 0.90]	0.76 [0.70, 0.82]
		4	0.82 [0.78, 0.86]	0.82 [0.76, 0.88]	0.79 [0.74, 0.84]	0.82 [0.75, 0.89]	0.85 [0.77, 0.93]	0.77 [0.71, 0.83]
		Δ	0.02 [−0.02, 0.05]	0.01 [−0.01, 0.03]	0.01 [−0.02, 0.03]	−0.01 [−0.03, 0.02]	0.03 [−0.01, 0.06]	0.01 [−0.01, 0.03]
	Time to peak growth, hour	0	0.11 [−0.02, 0.23]	0	0.05 [−0.06, 0.16]	0.05 [−0.04, 0.14]	0.17 [−0.08, 0.43]	0.08 [−0.09, 0.26]
		4	0.16 [−0.03, 0.34]	0.08 [−0.06, 0.21]	0.04 [−0.04, 0.12]	0	0.04 [−0.05, 0.13]	0.18 [−0.10, 0.47]
		Δ	0.05 [−0.15, 0.25]	0.08 [−0.06, 0.21]	−0.01 [−0.04, 0.02]	−0.05 [−0.14, 0.04]	−0.13 [−0.40, 0.14]	0.10 [−0.03, 0.23]
*Salmonella typhimurium*	Max OD index	0	0.77 [0.75, 0.80]	0.77 [0.74, 0.80]	0.78 [0.74, 0.82]	0.77 [0.74, 0.81]	0.79 [0.76, 0.82]	0.76 [0.72, 0.79]
		4	0.76 [0.72, 0.80]	0.76 [0.72, 0,79]	0.75 [0.72, 0.79]	0.77 [0.73, 0.80]	0.73 [0.66, 0.80]	0.75 [0.71, 0.80]
		Δ	−0.01 [−0.04, 0.01]	−0.01 [−0.05, 0.03]	−0.02 [−0.06, 0.01]	−0.01 [−0.04, 0.02]	−0.061 [−0.124, 0.001]	0.00 [−0.04, 0.04]
	Peak growth rate index	0	0.60 [0.59, 0.62]	0.61 [0.58, 0.64]	0.61 [0.59, 0.63]	0.63 [0.61, 0.65]	0.61 [0.60, 0.63]	0.61 [0.59, 0.62]
		4	0.61 [0.59, 0.62]	0.65 [0.61, 0.69]	0.62 [0.59, 0.65]	0.62 [0.60, 0.65]	0.64 [0.61, 0.66]	0.62 [0.60, 0.65]
		Δ	0.003 [−0.01, 0.02]	0.04 [0.01, 0.08]	0.01 [−0.02, 0.03]	−0.002 [−0.02, 0.02]	0.02 [0.00, 0.05]	0.017 [−0.003, 0.04]
	Time to peak growth, hour	0	2.20 [1.34, 3.06]	2.21 [1.17, 3.25]	2.51 [1.31, 3.71]	1.74 [0.87, 2.61]	2.96 [1.82, 4.10]	2.73 [1.94, 3.52]
		4	1.84 [1.09, 2.59]	1.30 [0.65, 1.94]	2.08 [0.95, 3.21]	1.13 [0.50, 1.76]	1.89 [0.97, 2.81]	2.45 [1.42, 3.48]
		Δ	−0.36 [−0.93, 0.21]	−0.91 [−1.88, 0.06]	−0.43 [−1.30, 0.45]	−0.60 [−1.61, 0.40]	−1.07 [−2.05, −0.09]	−0.28 [−1.18, 0.63]

ANOVA results: Pairwise comparisons (FS daily vs. placebo, IHAT vs. placebo, ASP vs. placebo, IHAT vs. FS daily, ASP vs. FS daily) and (FS daily vs. FS + MNP, FS daily vs. weekly) of Δ values showed no significant differences (Tukey HSD adjusted *p* values > 0.05) in a linear mixed-effects model adjusted for age, sex, and BMI.

1 Arithmetic means and 95% confidence intervals.

2 Δ = wk4–wk0, paired difference.

3 Ratio of slope of the growth curves (log OD vs. time [hours]) at the steepest point of the exponential growth phase to that of control.

4 Cells with a zero value indicate that all samples had an estimate of time to peak growth of 0 h.

**Table 4:** Phase I results: markers of gut inflammation.

Parameter	Week	Treatment					
		Placebo (*n* = 26)	FeSO_4_ 60 mg Fe/d (*n* = 24)	IHAT 60 mg Fe/d (*n* = 26)	ASP 60 mg Fe/d (*n* = 25)	FeSO_4_ 60 mg Fe/d + MNP (*n* = 25)	FeSO_4_ 420 mg Fe weekly (*n* = 25)
		Mean^1^ [95% CI]					
Fecal calprotectin, μg/g fresh wt	0	2.35 [1.56, 3.54]	1.56 [0.97, 2.51]	2.99 [1.90, 4.69]	2.58 [1.36, 4.87]	2.87 [1.87, 4.41]	1.68 [1.18. 2.39]
	4	1.79 [1.15, 2.78]	1.70 [1.10, 2.65]	2.36 [1.64, 3.38]	2.58 [1.44, 4.63]	2.64 [1.75, 3.98]	1.81 [1.24, 2.65]
	Δ^2^	0.76 [0.44, 1.30]	1.09 [0.79, 1.52]	0.79 [0.50, 1.26]	1.00 [0.65, 1.55]	0.92 [0.59, 1.43]	1.08 [0.75,1.56]
FIT, *n* (%) positive participants	0	0 (0)	0 (0)	0 (0)	0 (0)	2 (8)	1 (4)
	4	0 (0)	0 (0)	1 (4)	1 (4)	0 (0)	1 (4)
Fecal myeloperoxidase, μg/g	0	0.043 [0.021, 0.089]	0.030 [0.014, 0.068]	0.051 [0.026, 0.097]	0.061 [0.031, 0.118]	0.040 [0.022, 0.073]	0.025 [0.012, 0.053]
	4	0.033 [0.018, 0.061]	0.030 [0.014, 0.061]	0.037 [0.022, 0.064]	0.056 [0.026, 0.121]	0.040 [0.024, 0.067]	0.016 [0.008, 0.030]
	Δ	0.77 [0.49, 1.21]	0.97 [0.54, 1.75]	0.76 [0.49, 1.18]	0.93 [0.58, 1.47]	1.01 [0.63, 1.63]	0.63 [0.36, 1.11]
Fecal α_1−_antitrypsin, μg/g	0	2.23 [1.86, 2.67]	2.28 [2.04, 2.56]	1.75 [1.20, 2.54]	1.73 [1.28, 2.33]	1.90 [1.53, 2.37]	1.95 [1.55, 2.46]
	4	2.23 [1.95, 2.56]	2.22 [1.97, 2.51]	1.90 [1.46, 2.47]	1.78 [1.34, 2.36]	1.90 [1.45, 2.51]	2.19 [1.85, 2.58]
	Δ	1.00 [0.88, 1.14]	0.97 [0.90, 1.05]	1.09 [0.83, 1.42]	1.03 [0.86, 1.23]	1.00 [0.85, 1.18]	1.12 [0.97, 1.30]

ANOVA results: pairwise comparisons (FS daily vs. placebo, IHAT vs. placebo, ASP vs. placebo, IHAT vs. FS daily, ASP vs. FS daily) and (FS daily vs. FS + MNP, FS daily vs. weekly) of Δ values showed no significant differences (Tukey HSD adjusted *p* values > 0.05) in a linear mixed-effects model adjusted for age, sex, and BMI.

1 Geometric means and 95% confidence intervals based on log-transformed data.

2 Δ = wk 4/wk 0, paired ratios.

#### 3.1.3. Secondary endpoints

- **Iron Status**. No form of supplemental Fe significantly affected any parameter of iron status assessed in fasting blood samples over the 4-week intervention period (Table 5). The adjusted mean difference between ASP and placebo was 0.17 g/dl [95% CI: −0.28, 0.61]. The four-week change in fasting serum iron was not significantly different for FS weekly compared to FS daily, with an adjusted mean difference of 23.2 μg/dl [95% CI: −15.4, 61.8].
- **Gastrointestinal Symptoms**. Most (81%) participants reported at least one symptom, which were mostly of mild intensity and inconvenience. The leading symptom was change in stool color, reported by 69% of participants, followed by abnormal number of bowel movements (38%) and constipation (24%) (Table 6). The frequencies of these symptoms did not differ between iron treatments except for abdominal pain (omnibus test for IHAT, ASP, FS daily, and placebo, *p* = 0.008); stool color darkening (omnibus test for IHAT, ASP, FS daily, and placebo, *p* = 0.006); nausea (omnibus test for FS weekly, FS daily and FS + MNP, *p* = 0.009); and abnormal number of bowel movements (omnibus test for FS weekly, FS daily and FS + MNP, *p* = 0.010). Upon review by the study physician (JBM), the few instances of FIT positivity were not found to have corresponding anemia or abdominal pain.

**Table 5:** Phase I results: parameters of iron status.

Parameter	Week	Treatment					
		Placebo (*n* = 26)	FS daily 60 mg Fe/d (*n* = 24)	IHAT 60 mg Fe/d (*n* = 26)	ASP 60 mg Fe/d (*n* = 24)	FS + MNP 60 mg Fe/d (*n* = 25)	FS weekly 420 mg Fe (*n* = 25)
		Mean [95% CI]^1^					
Hemoglobin, g/dl	0	14.3 [13.7, 14.8]	13.9 [13.5, 14.3]	13.8 [13.3, 14.3]	14.1 [13.5, 14.7]	13.9 [13.5, 14.3]	13.6 [13.2, 14.0]
	4	13.9 [13.4, 14.4]	13.7 [13.2, 14.1]	13.6 [13.3, 14.1]	13.9 [13.4, 14.5]	13.6 [13.3, 14.0]	13.3 [12.9, 13.8]
	Δ^2^	−0.36 [−0.57, −0.15]	−0.24 [−0.54, 0.06]	−0.20 [−0.50, 0.09]	−0.16 [−0.34, 0.02]	−0.29 [−0.56, −0.03]	−0.24 [−0.52, 0.05]
Serum Fe, μg/dl	0	123 [108, 137]	122 [10, 138]	118 [105, 131]	102 [89, 115]	117 [101, 133]	111 [95, 127]
	4	108 [96, 120]	100 [85, 115]	104 [91, 116]	98 [86, 110]	105 [90, 119]	105 [90, 119]
	Δ	−14.6 [−26.1, −3.2]	−21.8 [−38.4, −5.2]	−14.4 [−28.0, −0.7]	−3.84 [−16.1, 8.5]	−12.3 [−28.8, 4.2]	−6.4 [−26.1, 13.3]
Serum transferrin, mg/dl	0	272 [257, 286]	259 [240, 278]	272 [253, 290]	257 [242, 271]	267 [254, 280]	255 [237, 273]
	4	267 [250, 283]	260 [246, 275]	277 [257, 294]	255 [240, 269]	262 [245, 278]	246 [231, 262]
	Δ	−5.1 [−12.8, 2.6]	1.4 [−15.2, 18.0]	4.8 [−7.5, 17.1]	−2.3 [−10.0, 5.3]	−5.2 [−14.4, 4.0]	−8.5 [−15.5, −1.5]
Transferrin sat’n, %	0	33.9 [29.6, 38.2]	33.5 [29.2, 37.9]	33.1 [29.4, 36.8]	29.8 [26.3, 33.3]	32.5 [28.5, 36.6]	32.2 [27.2, 37.2]
	4	30.7 [26.9, 34.6]	28.5 [24.0, 33.0]	28.5 [25.2, 31.9]	28.6 [25.4, 31.8]	29.9 [25.6, 34.2]	31.1 [26.9, 35.2]
	Δ	−3.2 [−6.5, 0.2]	−5.0 [−9.2, −0.9]	−4.5 [−8.2, −0.9]	−1.2 [−4.4, 2.1]	−2.6 [−7.2, 2.0]	−1.1 [−7.4, 5.2]
Serum ferritin, ng/dl	0	110 [80, 139]	128 [90, 165]	88 [70, 105]	114 [78, 150]	129 [78, 181]	120 [81, 158]
	4	101 [74, 128]	113 [78, 148]	83 [65, 102]	107 [76, 139]	101 [72, 130]	121 [83, 159]
	Δ	−9.0 [−21.5, 3.5]	−14.3 [−26.5, −2.2]	−4.4 [−14.5, 5.6]	−7.0 [−16.4, 2.4]	−28.2 [−61.5, 5.0]	1.0 [−11.6, 13.5]
Unsaturated Fe-binding capacity, μg/dl	0	238 [216, 261]	236 [215, 257]	240 [215, 265]	235 [218, 252]	235 [219, 252]	231 [207, 256]
	4	246 [221, 271]	248 [226, 269]	256 [233, 278]	240 [222,258]	242 [220, 264]	226 [207, 244]
	Δ	7.7 [−8.8, 24.3]	11.4 [−2.7, 25.5]	16.0 [−0.2,32.3]	5.2 [−6.8. 17.3]	6.6 [−10.2, 23.4]	−5.6 [−25.2, 13.9]
Total Fe-binding capacity, μg/dl	0	361 [342, 380]	358 [339, 377]	358 [336, 379]	337 [318, 356]	352 [336, 369]	342 [319, 366]
	4	354 [333, 376]	347 [329, 365]	359 [334, 384]	339 [319, 358]	347 [326, 368]	330 [312, 349]
	Δ	−6.9 [−17.3, 3.6]	−10.4 [−23.8, 3.0]	1.7 [−13.8, 17.2]	1.4 [−10.3, 13.1]	−5.7 [−17.6, 6.1]	−12.1 [−22.8, −1.4]

ANOVA results: Pairwise comparisons (FS daily vs. placebo, IHAT vs. placebo, ASP vs. placebo, IHAT vs. FS daily, ASP vs. FS daily) and (FS daily vs. FS + MNP, FS daily vs. weekly) of Δ values showed no significant differences (Tukey HSD adjusted *p* values > 0.05) in a linear mixed-effects model adjusted for age, sex, and BMI.

1 Arithmetic means and 95% confidence intervals.

2 Δ = wk4–wk0, paired difference.

**Table 6:** Phase I results: participants reporting symptoms at any weekly contact.

Symptom	Treatment group						
	Placebo (*n* = 26)	FS daily 60 mg Fe (*n* = 23)	IHAT 60 mg Fe/d (*n* = 26)	ASP 60 mg Fe/d (*n* = 26)	FS + MNP 60 mg Fe/d (*n* = 25)	FS weekly 420 mg Fe (*n* = 25)	Total (*n* = 151)
Nausea	2 (8%)	0 (0%)	1 (4%)	2 (8%)	0 (0%)	5 (20%)	10 (7%)
Vomiting	0 (0%)	0 (0%)	0 (0%)	0 (0%)	0 (0%)	0 (0%)	0 (0%)
Heartburn	2 (8%)	2 (9%)	3 (12%)	0 (0%)	3 (12%)	5 (20%)	15 (10%)
Abdominal pain	2 (8%)	1 (4%)	7 (27%)	0 (0%)	2 (8%)	4 (16%)	16 (11%)
Headache	4 (15%)	4 (17%)	7 (27%)	5 (19%)	3 (12%)	5 (20%)	28 (19%)
Out of breath	1 (4%)	1 (4%)	0 (0%)	3 (12%)	2 (8%)	0 (0%)	7 (5%)
Diarrhea	4 (15%)	2 (9%)	5 (19%)	0 (0%)	0 (0%)	4 (16%)	15 (10%)
Constipation	3 (12%)	8 (35%)	7 (27%)	5 (19%)	7 (28%)	6 (24%)	36 (24%)
Abnormal number of bowel movements	9 (35%)	12 (52%)	16 (62%)	9 (35%)	3 (12%)	9 (36%)	58 (38%)
Stool color change	8/22 (36%)	18/22 (82%)	19/24 (79%)	13/22 (59%)	16/23 (70%)	20/23 (87%)	94/136 (69%)

Fisher’s exact test was used to compare symptom reporting, among new iron forms and among modes of administration. The following significant differences were noted: abdominal pain, omnibus test for IHAT, ASP, FS daily, and placebo (*p* = 0.008); stool color change: omnibus test for IHAT, ASP, FS daily, and placebo (*p* = 0.006); nausea: omnibus test for FS weekly, FS daily and FS + MNP (*p* = 0.009); abnormal number of bowel movements: omnibus test for FS weekly, FS daily and FS + MNP (*p* = 0.010).

#### 3.1.4. Adverse events

Two participants reported adverse events. One participant, assigned to the placebo, was withdrawn from the study due to discomfort in taking the placebo. Another, receiving ASP, experienced diarrhea on intervention days 8 and 9, although that participant’s report on the Gut Irritation Questionnaire on day 8 was normal. Out of an abundance of caution, that participant was dropped from the study.

### 3.2. Results of phase II

#### 3.2.1. Volunteers

The Phase II recruitment scheme is shown in Figure 2. This produced a cohort of 86 volunteers comprised of mostly college-educated females and males aged 50–80 years (Table 7), recruited between June 2019 and October 2021. A total of 61 of these had also participated in Phase I but had completed that phase at least 1-year prior to enrolling in Phase II. During the study, participants consumed ca. 2,169 kcal/day, including ca. 85 g/day of protein more than half of which was from foods of animal origin. Their estimated dietary iron intakes were 14–16 mg/day (Table 7). Of the 79 participants that completed the study, 77 reported the number of returned capsules. All participants reported taking >80% of the allocated capsules during the study.

**Table 7:** Baseline characteristics of participants in Phase II.

Characteristic^1^	FS 120 mg Fe/d (*n* = 28)	IHAT 120 mg Fe/d (*n* = 24)	ASP 120 mg Fe/d (*n* = 27)
Age, y			
Mean (SD)	65.8 (6.2)	66.2 (7.3)	64.5 (6.9)
Range	54–80	51–79	50–80
Sex			
Female, *n* (%)	13 (46%)	11 (46%)	11 (41%)
BMI, kg/m^2^			
Mean (SD)	26.5 (4.2)	26.4 (3.39)	25.0 (3.7)
Range	20–34	22–34	19–35
Race			
White/Caucasian, *n* (%)	22 (79%)	15 (62%)	24 (89%)
Other, *n* (%)	6 (21%)	9 (38%)	3 (11%)
Education completed			
<4 y college, *n* (%)	3 (11%)	4 (17%)	9 (33%)
4 y college, *n* (%)	15 (54%)	12 (50%)	6 (22%)
Grad school or more, *n* (%)	10 (36%)	8 (33%)	12 (44%)
Dietary intake, median (IQR^2^)			
Total energy intake, kcal/d	2,132 (1,686, 2,432)	2,309 (1,649, 2,609)	2065 (1,548, 2,337)
Total protein intake, g/d	89 (65, 106)	89 (60, 106)	78 (67, 106)
% Animal protein	61 (51, 67)	59 (51, 68)	60 (42, 70)
% Plant protein	39 (33, 49)	42 (32, 49)	36 (30, 58)
Iron intake, mg/d	15 (11, 19)	16 (13, 20)	14 (11, 18)

1 Sample sizes due to missing data are indicated for individual variables.

2 Interquartile range.

#### 3.2.2. Primary endpoints

- ***Ex vivo* malarial invasion**. No form of iron administered at the level of 120 mg Fe/day affected the susceptibility of participant erythrocytes to *P. falciparum* infection (Table 8).
- ***Ex vivo* bacterial proliferation potential**. No form of iron administered at the level of 120 mg Fe/day affected the proliferation of any bacterium in participant plasma (Table 9).
- **Gut inflammation**. No form of Fe affected the 4-week changes in markers of gut inflammation (Table 10).

**Table 8:** Phase II results: *Ex vivo* malarial invasion of participant erythrocytes.

Week	FS 120 mg Fe/d (*n* = 28)	IHAT 120 mg Fe/d (*n* = 23)	IHAT 120 mg Fe/d (*n* = 23)
	Parasitemia % ratio (24 h/0 h), mean [95% CI]^1^		
0	1.88 [1.60, 2.21]	2.2 [1.82, 2.65]	1.98 [1.62, 2.40]
4	2.01 [1.73, 2.34]	2.19 [1.89, 2.53]	1.97 [1.65, 2.35]
Δ^2^	1.07 [0.94, 1.21]	0.99 [0.88, 1.12]	1.00 [0.91, 1.09]

ANOVA results: group comparisons (FS vs. IHAT, FS vs. ASP, IHAT vs. ASP) of wk0, wk4 and Δ values showed no significant differences (Tukey HSD adjusted *p* values > 0.05) in a linear model adjusted for age, sex, and BMI.

1 Geometric means and 95% confidence intervals based on log-transformed data.

2 Δ = wk4–wk0, paired ratios (for log-transformed data).

**Table 9:** Phase II results: *Ex vivo* proliferation of bacteria in presence of participants’ plasma.

Bacterium	Growth parameter	Week	FS 120 mg Fe/d (*n* = 27)	IHAT 120 mg Fe/d (*n* = 23)	ASP 120 mg Fe/d (*n* = 23)
			Means^1^ [95% CI]		
*Escherichia coli*	Max OD index	0	0.44 [0.38, 0.50]	0.43 [0.37, 0.49]	0.38 [0.32, 0.44]
		4	0.41 [0.36, 0.46]	0.41 [0.35, 0.48]	0.38 [0.32, 0.45]
		Δ^2^	−0.03 [−0.076, 0.016]	−0.017 [−0.067, 0.033]	0.001 [−0.059, 0.060]
	Peak growth rate index	0	0.68 [0.63, 0.72]	0.68 [0.63, 0.73]	0.65 [0.60, 0.70]
		4	0.67 [0.63, 0.72]	0.69 [0.63, 0.75]	0.65 [0.61, 0.70]
		Δ	−0.003 [−0.013, 0.008]	0.010 [−0.016, 0.036]	0.007 [−0.007, 0.021]
	Time to peak growth, hour^3^	0	0.062 [−0.013, 0.137]	0.04 [−0.04, 0.12]	0.031 [−0.034, 0.096]
		4	0.049 [−0.016, 0.114]	0	0.040 [−0.043, 0.124]
		Δ	−0.013 [−0.039, 0.013]	−0.038 [−0.117, 0.041]	0.009 [−0.010, 0.028]
*Acinetobacter baumannii*	Max OD index	0	0.76 [0.72, 0.79]	0.76 [0.72, 0.81]	0.72 [0.67, 0.77]
		4	0.75 [0.72, 0.78]	0.77 [0.74, 0.80]	0.75 [0.70, 0.79]
		Δ	−0.002 [−0.034, 0.030]	0.006 [−0.040, 0.052]	0.028 [−0.014, 0.070]
	Peak growth rate index	0	0.71 [0.68, 0.74]	0.74 [0.71, 0.77]	0.69 [0.66, 0.72]
		4	0.71 [0.68, 0.74]	0.74 [0.71, 0.77]	0.70 [0.67, 0.72]
		Δ	0.00 [−0.005, 0.004]	0.002 [−0.006, 0.009]	0.004 [−0.002, 0.011]
	Time to peak growth, hour	0	7.80 [7.32, 8.28]	7.98 [7.44, 8.52]	7.74 [7.17, 8.31]
		4	7.83 [7.42, 8.23]	8.05 [7.63, 8.48]	7.61 [7.05, 8.16]
		Δ	0.025 [−0.335, 0.385]	0.074 [−0.420, 0.568]	−0.134 [−0.421, 0.153]
*Klebsiella pneumoniae*	Max OD index	0	0.21 [0.17, 0.24]	0.20 [0.16, 0.24]	0.18 [0.15, 0.21]
		4	0.20 [0.17, 0.23]	0.18 [0.15, 0.21]	0.19 [0.16, 0.22]
		Δ	−0.005 [−0.032, 0.022]	−0.019 [−0.043, 0.006]	0.003 [−0.017, 0.023]
	Peak growth rate index	0	−0.475 [0.463, 0.486]	0.482 [0.466, 0.497]	0.468 [0.461, 0.474]
		4	0.474 [0.462, 0.486]	0.483 [0.468, 0.497]	0.469 [0.461, 0.476]
		Δ	−0.001 [−0.004, 0.003]	0.001 [−0.005, 0.007]	0.001 [−0.001, 0.004]
	Time to peak growth, hour	0	3.24 [2.34, 4.13]	2.86 [2.07, 3.66]	3.21 [2.25, 4.17]
		4	3.17 [2.49, 3.84]	2.55 [1.74, 3.36]	3.34 [2.46, 4.22]
		Δ	−0.072 [−0.799, 0.656]	−0.312 [−0.928, 0.303]	0.127 [−0.728, 0.983]
*Staphylococcus aureus*	Max OD index	0	0.68 [0.61, 0.74]	0.69 [0.61, 0.77]	0.75 [0.69, 0.81]
		4	0.70 [0.64, 0.76]	0.69 [0.60, 0.78]	0.75 [0.69, 0.81]
		Δ	0.023 [−0.008, 0.055]	0.000 [−0.059, 0.058]	0.001 [−0.035, 0.037]
	Peak growth rate index	0	0.94 [0.88, 1.01]	0.92 [0.84, 1.00]	0.77 [0.72, 0.83]
		4	0.94 [0.87, 1.01]	0.91 [0.82, 0.99]	0.79 [0.74, 0.84]
		Δ	0.005 [−0.020, 0.030]	−0.007 [−0.052, 0.038]	0.016 [−0.012, 0.044]
	Time to peak growth, hour	0	0.19 [−0.03, 0.42]	0	0.14 [−0.05, 0.33]
		4	0.07 [−0.05, 0.18]	0	0.03 [−0.03, 0.09]
		Δ	−0.126 [−0.313, 0.062]	0	−0.109 [−0.314, 0.097]
*Salmonella typhimurium*	Max OD index	0	0.71 [0.66, 0.75]	0.68 [0.61, 0.76]	0.69 [0.64, 0.74]
		4	0.70 [0.65, 0.74]	0.69 [0.65, 0.74]	0.71 [0.64, 0.78]
		Δ	−0.010 [−0.042, 0.023]	0.012 [−0.057, 0.081]	0.016 [−0.049, 0.080]
	Peak growth rate index	0	0.613 [0.0600, 0.626]	0.618 [0.596, 0.641]	0.611 [0.589, 0.633]
		4	0.606 [0.592, 0.621]	0.608 [0.593, 0.624]	0.626 [0.593, 0.659]
		Δ	−0.007 [−0.017, 0.003]	−0.010 [−0.028, 0.009]	0.015 [−0.012, 0.042]
	Time to peak growth, hourr	0	3.58 [2.46, 4.69]	4.00 [2.70, 5.29]	4.08 [3.00, 5.15]
		4	3.83 [2.83, 4.82]	3.89 [2.95, 4.82]	3.91 [2.90, 4.93]
		Δ	0.253 [−0.554, 1.061]	−0.113 [−0.859, 0.632]	−0.160 [−1.039, 0.719]

ANOVA results: Pairwise comparisons (FS vs. IHAT, FS vs. ASP, IHAT vs. ASP) of Δ values showed no significant differences (Tukey HSD adjusted *p* values > 0.05) in a linear mixed-effects model adjusted for age, sex, and BMI for each growth parameter.

1 Arithmetic means and 95% confidence intervals.

2 Δ = wk4–wk0, paired difference.

3 Cells with a zero indicate that all samples had an estimate of time to peak growth of 0 h.

**Table 10:** Phase II results: markers of gut inflammation.

Parameter	Week	Treatment		
		FeSO_4_ 120 mg Fe/d (*n* = 28)	IHAT 120 mg Fe/d (*n* = 23)	ASP 120 mg Fe/d (*n* = 26)
		Means [95% CI]^1^		
Fecal calprotectin, μg/g fresh wt	0	1.61 [1.03, 2.52]	1.66 [1.15, 2.39]	1.82 [1.21, 2.73]
	4	1.02 [0.66, 1.58]	1.17 [0.73, 1.89]	1.24 [0.82, 1.89]
	Δ^2^	0.63 [0.38, 1.07]	0.71 [0.52, 0.96]	0.68 [0.47, 0.98]
FIT, *n* (%) positive participants	0	0 (0)	0 (0)	0 (0)
	4	0 (0)	0 (0)	0 (0)
Fecal myeloperoxidase, μg/g	0	0.039 [0.021, 0.072]	0.028 [0.015, 0.051]	0.027 [0.013, 0.056]
	4	0.041 [0.017, 0.097]	0.023 [0.013, 0.041]	0.024 [0.012, 0.048]
	Δ	1.06 [0.46, 2.41]	0.82 [0.52, 1.31]	0.89 [0.56, 1.42]
Fecal α_1−_antitrypsin, μg/g	0	4.82 [3.10, 7.49]	5.99 [3.87, 9.27]	4.00 [2.24, 7.14]
	4	4.67 [2.88, 7.58]	5.49 [3.69, 8.16]	4.51 [2.63, 7.71]
	Δ	0.97 [0.79, 1.20]	0.92 [0.75, 1.12]	1.13 [0.95, 1.34]

ANOVA results by group comparisons (FeSO_4_ vs. IHAT, FeSO_4_ vs. ASP, IHAT vs. ASP) of Δ values showed no significant differences (Tukey HSD adjusted *p* values > 0.05) in a linear model adjusted for age, sex, and BMI.

1 Geometric means and 95% confidence intervals based on log-transformed data.

2 Δ = wk4/wk0, paired ratios (for log-transformed data).

#### 3.2.3. Secondary endpoints

- **Iron Status**. No form of supplemental Fe significantly affected 4-week changes in any parameter of iron status assessed in fasting blood samples, including transferrin saturation, which remained 30–40% (Table 11). The four-week changes in transferrin saturation for between ASP and FS (adjusted mean difference of 4.32% [95% CI: −3.82, 12.46]), and between ASP and IHAT (adjusted mean difference of 4.24% IHAT [95% CI: −4.23, 12.72]) were not significantly different.
- **Gastrointestinal Symptoms**. Most (87%) participants reported at least one symptom, which were mostly of mild intensity and inconvenience. The most frequently reported symptom was stool color change (86% of participants), abnormal number of bowel movements (32%), and constipation (27%) (Table 12). The presence of all symptoms declined in the last (4th) week. There was no significant difference in the frequency of reporting of any symptom between the intervention groups.

**Table 11:** Phase II results: parameters of iron status.

Parameter	Week	FS 120 mg Fe/d (*n* = 28)	IHAT 120 mg Fe/d (*n* = 23)	ASP 120 mg Fe/d (*n* = 26)
		Means^1^ [95% CI]		
Hemoglobin, g/dl	0	14.1 [13.7, 14.5]	14.1 [13.5, 14.6]	14.2 [13.7,14.6]
	4	14.0 [13.6, 14.5]	13.8 [13.2, 14.3]	14.2 [13.7, 14.6]
	Δ^2^	−0.1 [−0.3, 0.1]	−0.3 [−0.5, −0.1]	0.0 [−0.2, 0.2]
Serum Fe, μg/dl	0	112 [101, 124]	104 [92, 117]	92 [78, 105]
	4	107 [92, 122]	99 [85, 114]	102 [88, 115]
	Δ	−5.6 [−22.7, 11.6]	−5.3 [−20.8, 10.2]	10.0 [−7.1, 27.1]
Serum transferrin, mg/dl	0	250 [234, 267]	257 [238, 277]	249 [240, 259]
	4	242 [232, 252]	252 [235, 269]	252 [241, 263]
	Δ	−8.3 [−21.7, 5.2]	−5.6 [−13.1, 2.0]	2.9 [−6.4, 12.1]
Transferrin saturation, %	0	34.1 [30.1, 38.2]	31.1 [26.9, 35.4]	27.3 [23.6, 30.9]
	4	32.8 [28.4, 37.2]	29.7 [25.6, 33.9]	30.0 [26.5, 33.5]
	Δ	−1.3 [−6.1, 3.4]	−1.4 [−6.2, 3.5]	2.7 [−1.9, 7.2]
Serum ferritin, ng/dl	0	102 [77, 127]	95 [58, 131]	94 [70, 117]
	4	102 [77, 127]	84 [56, 111]	91 [70, 112]
	Δ	0.1 [−8.6, 8.9]	−11.0 [−23.5, 1.4]	−2.6 [−12.6, 7.4]
Unsaturated Fe-binding capacity, μg/dl	0	222 [197, 248]	236 [209, 262]	240 [226, 255]
	4	217 [199, 235]	234 [212, 256]	235 [219, 251]
	Δ	−5.1 [−25.4, 15.1]	−1.8 [−17.6, 14.1]	−4.9 [−21.1, 11.2]
Total Fe-binding capacity, μg/dl	0	334 [314, 355]	340 [318, 363]	332 [319, 344]
	4	324 [311, 336]	333 [313, 353]	337 [321, 352]
	Δ	−10.7 [−28.5, 7.1]	−7.1 [−16.6, 2.4]	5.0 [−8.5, 18.5]

ANOVA results: pairwise comparisons (FS vs. IHAT, FS vs. ASP, IHAT vs. ASP) of Δ values showed no significant differences (Tukey HSD adjusted *p* values > 0.05) in a linear mixed-effects model adjusted for age, sex, and BMI.

1 Arithmetic means and 95% confidence intervals.

2 Δ = wk4–wk0, paired differences.

**Table 12:** Phase II results: participants reporting symptoms at any weekly contact.

Symptom	Treatment group			
	FS 120 mg Fe daily (*n* = 28)	IHAT 120 mg Fe/d (*n* = 23)	ASP 120 mg Fe/d (*n* = 26)	Total (*n* = 77)
Nausea	5 (18%)	3 (13%)	0 (0%)	8 (10%)
Vomiting	0 (0%)	1 (4%)	0 (0%)	1 (1%)
Heartburn	3 (11%)	1 (4%)	1 (4%)	5 (6%)
Abdominal pain	0 (0%)	2 (9%)	0 (0%)	2 (3%)
Headache	2 (7%)	3 (13%)	3 (12%)	8 (10%)
Out of breath	0 (0%)	1 (4%)	0 (0%)	1 (1%)
Diarrhea	1 (4%)	2 (9%)	2 (8%)	5 (6%)
Constipation	7 (25%)	9 (39%)	5 (19%)	21 (27%)
Abnormal number of bowel movements	8 (29%)	8 (35%)	9 (35%)	25 (32%)
Stool color change	25 (89%)	20 (87%)	21 (81%)	66 (86%)

No significant differences were noted in the frequency of any reported symptom between any treatment groups.

#### 3.2.4. Adverse event

After 2 weeks of supplementation, one participant experienced moderate nausea 30–40 min of taking ASP. This was accompanied by constipation and irregular bowel movements. The supplement was discontinued, the participant was dropped from the study and was advised to contact the study physician. This participant did not participate in the Phase I study.

## 4. Discussion

The goal of the Safe Iron Study was to examine the safety of two novel forms of iron, IHAT and ASP, in comparison to that of FS at two levels of supplementation, 60 and 120 mg/day. In addition, the effects of daily vs. weekly FS dosing were compared, as were the effects of co-administration of MNP with FS.

### 4.1. Primary endpoints

- **Susceptibility to malarial infection**. The magnetic isolation of mature parasites yielded nearly homogeneous populations of schizonts without complications of uninfected erythrocytes. Schizont morphology, as examined microscopically, showed no effects of participant iron supplementation; merozoites released from purified schizonts were able to invade erythrocytes directly. That merozoites were also able to re-invade participant erythrocytes (data not shown), demonstrated normal development of parasites under these conditions. That no form or level of iron affected malarial invasion of participant erythrocytes (Tables 2, 8) suggests that iron supplementation of these iron-replete adults did not affect their apparent risk to clinical malaria infection. This finding is consistent with minimal effects on plasma NTBI, which has been proposed to increase the intensity of malarial infections (19, 20). Still, the possibility that the *ex vivo* assay may not fully reflect susceptibility to infection in clinical settings cannot be excluded. This study did not address the role in the malarial life cycle of hepatocytes in which schizonts are produced from sporozoites delivered by *Anopheles* injection into the bloodstream. Therefore, these results cannot be taken as dispositive of the hypothesis that iron supplementation of young iron-replete individuals can enhance clinical malarial infection as proposed by Clark et al. (62).
- **Bacterial proliferation potential**. The *ex vivo* bacterial growth assays performed in this study showed no significant effects of iron supplementation (Tables 3, 9). These findings differ from those of Cross et al. (59), who found oral supplementation of participants of marginal iron status with FS (2 mg/kg) to increase, within 4 h, the *ex vivo* growth of pathogenic bacteria (*E. coli*, *Yersinia enterocolitica*, *S. enterica* serovar *Typhimurium*, and *Staphylococcus epidermidis*) in participant sera. Those *ex vivo* responses were strongly correlated with increase in transferrin saturation in all cases except *Staphylococcus aureus*, which is known to scavenge heme iron and did not respond to participant iron supplementation. In the present study, no treatment effects were observed on transferrin saturation or any other parameter of iron status. Nevertheless, significant increases in max OD and peak growth rates of *E. coli, K. pneumoniae, S. aureus, S. typhimurium,* and *A. baumannii* were observed within 2 h of the first weekly dose of 420 mg Fe as FS. IHAT was shown to be noninferior to FS daily in both *E. coli* and *S. typhimurium*, and the other iron supplementation groups were shown to be noninferior to FS daily in *S. typhimurium*. Short-term responses were not observed for any treatment over the 4-week course of supplementation. Such null results are consistent with iron-replete participants having low enteric absorption of iron, as indicated by their null transferrin saturation responses in both phases of the study (Tables 5, 11). We did, however, note a significant difference in the 4-week changes in proliferation potential of *A. baumannii* for participants given ASP as compared to those given FS; however, that difference was noted only when assessed in PP samples (see Supplementary Data, Supplementary Table 1).
- **Gut inflammation**. Markers of gut inflammation revealed no significant impacts of iron supplementation on the gut inflammatory tone (Tables 4, 10), and noninferiority of IHAT was shown for fecal calprotectin. This suggest that unabsorbed iron from moderate to high levels of supplementation do not promote intestinal inflammation in healthy, iron-replete adults, and that markers of intestinal inflammation are not useful indicators clinical symptoms of abdominal pain and constipation that can be experienced with iron supplements.

### 4.2. Secondary endpoints

- **Iron status**. The participants in both phases of this study were iron-replete, as indicated by their normal hemoglobin levels, their estimated dietary iron intakes, which exceeded the recommended daily allowance (8 mg) (42), and their baseline values of biomarkers of iron status (Tables 5, 11). In no case did any form of supplemental iron, at either the 60 mg/day or 120 mg/day dose level, increase iron status (e.g., transferrin saturation remained ca. 30%). That no increase in serum iron or transferrin saturation was detected 2 h after iron dosing (data not shown), is as expected for iron-replete individuals within the timeframe of these studies, suggesting that absorption was low for all iron supplements and implying that iron treatment did not increase NTBI in the plasma (19) and that lower gut exposure to non-absorbed iron was significant.
- **Gastrointestinal symptoms**. Most participants reported changes in stool color (darkening); some reported abnormal number of bowel movements and/or constipation neither of which they considered more than mildly inconvenient (Tables 6, 12). In Phase I, participants receiving FS, IHAT or ASP more frequently reported stool color changes than the placebo group. Those receiving IHAT more frequently reported abdominal pain than the FS or ASP groups. Reports of abnormal bowel movements associated with daily supplementation with FS were fewer for participants also receiving MNP and those taking FS weekly in phase I; no treatment differences were observed in phase II. In Phase I abdominal pain was reported by 7 participants (27%) taking IHAT; however, in phase II, in which iron doses were doubled, abdominal pain was reported by only 2 participants (9%) both of whom were taking IHAT. The profile of reported gastrointestinal symptoms is consistent with that expected for oral iron supplements, while those associated with FS were lower than expected from other studies (65). Three participants were withdrawn from the study due to adverse events including abdominal pain, nausea, and constipation; as most of these cases involved non-specific symptoms, these low numbers may not be related to treatment.

While the Food and Nutrition Board (42) suggested 120 mg Fe/day as the LOAEL for FS, a more recent meta-analysis of more than 40 clinical trials (65) found that the prevalence of gastrointestinal complaints associated with FS administration appears to be idiosyncratic and not related to dose. Accordingly, the observation that, in Phase II of this study, neither IHAT nor ASP providing 120 mg iron/day produced a pattern of reported gastrointestinal symptoms different from FS is not surprising, as the insolubility of each in the post-duodenal gut would suggest that neither form has substantial interactions with those epithelial surfaces.

### 4.3. Strengths and limitations

This was a rigorously designed study that addressed the effects of multiple modalities of iron supplementation of iron-replete individuals on key aspects of malarial infectivity, bacterial infections, and gut inflammation. Both phases of the study were powered on these primary outcomes which, thus, had type I error control. Limitations include that we did not perform power calculations for other outcomes, i.e., iron status parameters, markers of gut inflammation besides fecal calprotectin, and GI side effects, or noninferiority tests. That iron supplementation did not affect malarial infection of erythrocytes does not imply that such treatment may not affect susceptibility to malarial infection in clinical settings, as we did not access effects on hepatocytes, which are also important in the cycle of malarial infection.

## 5. Conclusion

This study demonstrated that two novel forms of bioavailable iron, IHAT and ASP (at either dose level of 60 mg/day in Phase I or 120 mg/day in Phase II) for 28 days did not produce significant different responses in the primary outcomes: malaria infectivity, bacterial proliferation, and gut inflammation. Similarly, there were no significant differences in parameters of iron status between these forms, with or without micronutrient supplementation. The majority (69–87%) of participants experienced changes in stool color, with over 25% of participants reporting changes in stool number and constipation consistent with iron supplementation. At the 60 mg/day dose used in phase I, IHAT produced abdominal pain in 27% of participants; unexpectedly, no treatment-related difference in symptoms were observed at the therapeutic dose in phase II. Taken overall, these results indicate that IHAT and ASP may be safe and as well tolerated as FS in these healthy, iron-replete adults.

## Data availability statement

The raw data supporting the conclusions of this article will be made available by the authors, without undue reservation.

## Ethics statement

The studies involving human participants were reviewed and approved by the Tufts Health Sciences Institutional Review Board. The participants provided their written informed consent to participate in this study.

## Author contributions

GC, SM, EL, JM, JL, AC, DW, and KB designed the research. EL, EO, and MD coordinated the project. JM provided medical oversight. JL, MO, LM, and FN provided essential materials (bacterial cultures) and conducted bacterial proliferation studies. AC, CS, and AQ provided essential materials (malarial parasites) and conducted malarial infectivity studiers. CG conducted participant recruitment, dietary assessment, treatment administration, and sample collection. EO, GP, and WG conducted clinical analyses. KB, GM, and JW managed and analyzed the data. All authors contributed to the article and approved the submitted version.

## Footnotes

^1^ https://github.com/JuliaStats/StatsBase.jl

## References

[1] Hurrell R Egli I. Iron bioavailability and dietary reference values. Am J Clin Nutr. (2010) 91:1461S–7S. doi: 10.3945/ajcn.2010.28674F 20200263

[2] Jaeggi T Kortman GA Moretti D Chassard C Holding P Dostal A. Iron fortification adversely affects the gut microbiome, increases pathogen abundance and induces intestinal inflammation in Kenyan infants. Gut. (2015) 64:731–42. doi: 10.1136/gutjnl-2014-307720, PMID: 25143342

[3] Paganini D Uyoga MA Zimmermann MB. Iron fortification of foods for infants and children in low-income countries: effects on the gut microbiome, gut inflammation, and diarrhea. Nutrients. (2016) 8:494–505. doi: 10.3390/nu8080494, PMID: 27529276 PMC4997407

[4] Tang M Frank DN Sherlock L Ir D Robertson CE Krebs NF. Effect of vitamin E with therapeutic iron supplementation on iron repletion and gut microbiome in US Iron deficient infants and toddlers. J Pediatr Gastroenterol Nutr. (2016) 63:379–85. doi: 10.1097/MPG.0000000000001154, PMID: 27548249 PMC4994979

[5] Zimmermann MB Chassard C Rohner F N'Goran EK Nindjin C Dostal A. The effects of iron fortification on the gut microbiota in African children: a randomized controlled trial in cote d'Ivoire. Am J Clin Nutr. (2010) 92:1406–15. doi: 10.3945/ajcn.110.004564, PMID: 20962160

[6] Mayo-Wilson E Imdad A Junior J Dean S Bhutta ZA. Preventive zinc supplementation for children, and the effect of additional iron: a systematic review and meta-analysis. BMJ Open. (2014) 4:e004647. doi: 10.1136/bmjopen-2013-004647, PMID: 24948745 PMC4067863

[7] Soofi S Cousens S Iqbal SP Akhund T Khan J Ahmed I. Effect of provision of daily zinc and iron with several micronutrients on growth and morbidity among young children in Pakistan: a cluster-randomised trial. Lancet. (2013) 382:29–40. doi: 10.1016/S0140-6736(13)60437-7 23602230

[8] Barffour MA Schulze KJ Coles CL Chileshe J Kalungwana N Arguello M. High Iron Stores in the low Malaria Season Increase Malaria Risk in the high transmission season in a prospective cohort of rural Zambian children. J Nutr. (2017) 147:1531–6. doi: 10.3945/jn.117.250381, PMID: 28701387

[9] Gwamaka M Kurtis JD Sorensen BE Holte S Morrison R Mutabingwa TK. Iron deficiency protects against severe plasmodium falciparum malaria and death in young children. Clin Infect Dis. (2012) 54:1137–44. doi: 10.1093/cid/cis010, PMID: 22354919 PMC3309886

[10] Jonker FA Calis JC van Hensbroek MB Phiri K Geskus RB Brabin BJ. Iron status predicts malaria risk in Malawian preschool children. PLoS One. (2012) 7:e42670. doi: 10.1371/journal.pone.0042670, PMID: 22916146 PMC3420896

[11] Lynch S Stoltzfus R Rawat R. Critical review of strategies to prevent and control iron deficiency in children. Food Nutr Bull. (2007) 28:S610–20. doi: 10.1177/15648265070284S413, PMID: 18297898

[12] Moya-Alvarez V Cottrell G Ouedraogo S Accrombessi M Massougbodgi A Cot M. High Iron levels are associated with increased malaria risk in infants during the first year of life in Benin. Am J Trop Med Hyg. (2017) 97:497–503. doi: 10.4269/ajtmh.16-0001, PMID: 28722565 PMC5544062

[13] Nyakeriga AM Troye-Blomberg M Dorfman JR Alexander ND Back R Kortok M. Iron deficiency and malaria among children living on the coast of Kenya. J Infect Dis. (2004) 190:439–47. doi: 10.1086/422331, PMID: 15243915

[14] Portugal S Carret C Recker M Armitage AE Goncalves LA Epiphanio S. Host-mediated regulation of superinfection in malaria. Nat Med. (2011) 17:732–7. doi: 10.1038/nm.2368, PMID: 21572427 PMC4200394

[15] Sazawal S Black RE Ramsan M Chwaya HM Stoltzfus RJ Dutta A. Effects of routine prophylactic supplementation with iron and folic acid on admission to hospital and mortality in preschool children in a high malaria transmission setting: community-based, randomised, placebo-controlled trial. Lancet. (2006) 367:133–43. doi: 10.1016/S0140-6736(06)67962-2 16413877

[16] Dogan B Suzuki H Herlekar D Sartor RB Campbell BJ Roberts CL. Inflammation-associated adherent-invasive *Escherichia coli* are enriched in pathways for use of propanediol and iron and M-cell translocation. Inflamm Bowel Dis. (2014) 20:1919–32. doi: 10.1097/MIB.0000000000000183, PMID: 25230163

[17] Werner T Wagner SJ Martinez I Walter J Chang JS Clavel T. Depletion of luminal iron alters the gut microbiota and prevents Crohn's disease-like ileitis. Gut. (2011) 60:325–33. doi: 10.1136/gut.2010.216929, PMID: 21076126

[18] Prentice AM Doherty CP Abrams SA Cox SE Atkinson SH Verhoef H. Hepcidin is the major predictor of erythrocyte iron incorporation in anemic African children. Blood. (2012) 119:1922–8. doi: 10.1182/blood-2011-11-391219, PMID: 22228627 PMC3351093

[19] Hurrell R. Iron and malaria: absorption, efficacy and safety. Int J Vitam Nutr Res. (2010) 80:279–92. doi: 10.1024/0300-9831/a000035, PMID: 21462111

[20] Hurrell RF. Safety and efficacy of iron supplements in malaria-endemic areas. Ann Nutr Metab. (2011) 59:64–6. doi: 10.1159/000332140, PMID: 22123642 PMC7265417

[21] Kartikasari AE Georgiou NA Visseren FL van Kats-Renaud H van Asbeck BS Marx JJ. Endothelial activation and induction of monocyte adhesion by nontransferrin-bound iron present in human sera. FASEB J. (2006) 20:353–5. doi: 10.1096/fj.05-4700fje 16368718

[22] Pereira DI Aslam MF Frazer DM Schmidt A Walton GE McCartney AL. Dietary iron depletion at weaning imprints low microbiome diversity and this is not recovered with oral Nano Fe(III). Microbiology. (2015) 4:12–27. doi: 10.1002/mbo3.213, PMID: 25461615 PMC4335973

[23] US Food & drug GRAS Notices (2016). Available at: www.cfsanappsexternal.fda.gov/scripts/fdcc/index.cfm?set=GRASNotices&id=82.

[24] Codus Alimentarus International Food Standards (2019). Available at: www.fao.org › fao-who-codexalimentarius › sh-proxy.

[25] Reddy MB Armah SM. Impact of Iron-enriched aspergillus oryzae on Iron bioavailability, safety, and gut microbiota in rats. J Agric Food Chem. (2018) 66:6213–8. doi: 10.1021/acs.jafc.8b01758, PMID: 29852063

[26] Lewis ED Wu D Mason JB Chishti AH Leong JM Barger K. Safe and effective delivery of supplemental iron to healthy older adults: the double-blind, randomized, placebo-controlled trial protocol of the safe Iron study. Gates Open Res. (2019) 3:1510. doi: 10.12688/gatesopenres.13039.2, PMID: 33655197 PMC7890045

[27] Bries AE Wang C Agbemafle I Wels B Reddy MB. Assessment of acute serum Iron, non-transferrin-bound Iron, and gastrointestinal symptoms with 3-week consumption of Iron-enriched aspergillus oryzae compared with ferrous sulfate. Curr Dev Nutr. (2019) 3:nzz127. doi: 10.1093/cdn/nzz127, PMID: 32154497 PMC7053575

[28] Matuszek G. Lewis E. Safe and effective delivery of supplemental iron to healthy volunteers.

[29] Multiple Micronutrient Powder Supply and Outlook (2016).UNICEF Supply Div. Available at: https://unicef.org/media/4926/file/MNP-market-note-September-2016.pdf.

[30] Powell JJ Bruggraber SF Faria N Poots LK Hondow N Pennycook TJ. A nano-disperse ferritin-core mimetic that efficiently corrects anemia without luminal iron redox activity. Nanomedicine. (2014) 10:1529–38. doi: 10.1016/j.nano.2013.12.011, PMID: 24394211 PMC4315135

[31] Aslam MF Frazer DM Faria N Bruggraber SF Wilkins SJ Mirciov C. Ferroportin mediates the intestinal absorption of iron from a nanoparticulate ferritin core mimetic in mice. FASEB J. (2014) 28:3671–8. doi: 10.1096/fj.14-251520, PMID: 24776745 PMC4101650

[32] Latunde-Dada GO Pereira DI Tempest B Ilyas H Flynn AC Aslam MF. A nanoparticulate ferritin-core mimetic is well taken up by HuTu 80 duodenal cells and its absorption in mice is regulated by body iron. J Nutr. (2014) 144:1896–902. doi: 10.3945/jn.114.201715, PMID: 25342699 PMC4230207

[33] Pereira DI Bruggraber SF Faria N Poots LK Tagmount MA Aslam MF. Nanoparticulate iron(III) oxo-hydroxide delivers safe iron that is well absorbed and utilised in humans. Nanomedicine. (2014) 10:1877–86. doi: 10.1016/j.nano.2014.06.012 24983890 PMC4228177

[34] EFSAa Panel on Nutrition NF Food A Turck D Bohn T Castenmiller J De Henauw S. Safety of iron hydroxide adipate tartrate as a novel food pursuant to regulation (EU) 2015/2283 and as a source of iron in the context of directive 2002/46/EC. EFSA J. (2021) 19:e06935. doi: 10.2903/j.efsa.2021.6935, PMID: 34938369 PMC8662805

[35] Reddy MB Armah SM Stewart JW O'Brien KO. Iron absorption from Iron-enriched aspergillus oryzae is similar to ferrous sulfate in healthy female subjects. Curr Dev Nutr. (2018) 2:nzy004. doi: 10.1093/cdn/nzy004, PMID: 30019027 PMC6041945

[36] Bries AE Hurrell RF Reddy MB. Iron absorption from bouillon fortified with Iron-enriched aspergillus oryzae is higher than that fortified with ferric pyrophosphate in young women. J Nutr. (2020) 150:1109–15. doi: 10.1093/jn/nxaa035, PMID: 32073619 PMC7303367

[37] World Health Organization. WHO recommendations on antenatal Care for a Positive Pregnancy Experience. Geneva: EVIMalaR, Glasgow, UK (2016). 196 p. 28079998

[38] Bhatla N Kaul N Lal N Kriplani A Agarwal N Saxena R. Comparison of effect of daily versus weekly iron supplementation during pregnancy on lipid peroxidation. J Obstet Gynaecol Res. (2009) 35:438–45. doi: 10.1111/j.1447-0756.2008.00972.x, PMID: 19527380

[39] Haidar J Omwega AM Muroki NM Ayana G. Daily versus weekly iron supplementation 9nd prevention of iron deficiency anaemia in lactating women. East Afr Med J. (2003) 80:11–6. doi: 10.4314/eamj.v80i1.8661, PMID: 12755236

[40] Siddiqui IA Rahman MA Jaleel A. Efficacy of daily vs. weekly supplementation of iron in schoolchildren with low iron status. J Trop Pediatr. (2004) 50:276–8. doi: 10.1093/tropej/50.5.276, PMID: 15510758

[41] Troesch B Egli I Zeder C Hurrell RF Zimmermann MB. Fortification iron as ferrous sulphate plus ascorbic acid is more rapidly absorbed than as sodium iron EDTA but neither increases serum nontransferrin-bound iron in women. J Nutr. (2011) 141:822–7. doi: 10.3945/jn.110.136127 21430252

[42] Food and Nutrition Board (2001). Dietary reference intakes: For vitamin a, vitamin K, arsenic, boron, chromium, copper, iodine, Iron, manganese, molybdenum, nickel, silicon, vanadium and zinc. National Academy Press, Washington, DC. p 290–393. 25057538

[43] Moll K Ljungstrom I Perlmann H Scherf A Wahlgren H. Methods in malaria research. 6th ed EVIMalaR Glasgow, UK, and MR4/ATCC, Manassas, VA, USA. (2013). 474 p.

[44] Trager W Jensen JB. Human malaria parasites in continuous culture. Science. (1976) 193:673–5. doi: 10.1126/science.781840, PMID: 781840

[45] Ding Y Onodera Y Lee JC Hooper DC. NorB, an efflux pump in *Staphylococcus aureus* strain MW2, contributes to bacterial fitness in abscesses. J Bacteriol. (2008) 190:7123–9. doi: 10.1128/JB.00655-08, PMID: 18723624 PMC2580682

[46] Taylor TA Unakal CG. Staphylococcus Aureus. Edtion ed. Treasure Island (FL): StatPearls (2022).

[47] Geisinger E Mortman NJ Vargas-Cuebas G Tai AK Isberg RR. A global regulatory system links virulence and antibiotic resistance to envelope homeostasis in *Acinetobacter baumannii*. PLoS Pathog. (2018) 14:e1007030. doi: 10.1371/journal.ppat.1007030 29795704 PMC5967708

[48] Wong D Nielsen TB Bonomo RA Pantapalangkoor P Luna B Spellberg B. Clinical and pathophysiological overview of Acinetobacter infections: a century of challenges. Clin Microbiol Rev. (2017) 30:409–47. doi: 10.1128/CMR.00058-16, PMID: 27974412 PMC5217799

[49] Jorgensen MG van Raaphorst R Veening JW. Noise and stochasticity in gene expression: a pathogenic fate determinant. Methods Microbiol. (2013) 40:157. doi: 10.1016/B978-0-12-417029-2.00006-6

[50] Graham SM. Nontyphoidal salmonellosis in Africa. Curr Opin Infect Dis. (2010) 23:409–14. doi: 10.1097/QCO.0b013e32833dd25d 20736739

[51] Pulford CV Perez-Sepulveda BM Canals R Bevington JA Bengtsson RJ Wenner N. Stepwise evolution of *Salmonella Typhimurium* ST313 causing bloodstream infection in Africa. Nat Microbiol. (2021) 6:327–38. doi: 10.1038/s41564-020-00836-1, PMID: 33349664 PMC8018540

[52] Paczosa MK Silver RJ McCabe AL Tai AK McLeish CH Lazinski DW. Transposon mutagenesis screen of *Klebsiella pneumoniae* identifies multiple genes important for resisting antimicrobial activities of neutrophils in mice. Infect Immun. (2020) 88: 19. doi: 10.1128/IAI.00034-20, PMID: 31988174 PMC7093148

[53] Zander DS Farver CF. Pulmonary pathology. 2nd ed. Philadelphia, PA: Elsevier (2018). 974 p.

[54] Ciesielczuk H Doumith M Hope R Woodford N Wareham DW. Characterization of the extra-intestinal pathogenic *Escherichia coli* ST131 clone among isolates recovered from urinary and bloodstream infections in the United Kingdom. J Med Microbiol. (2015) 64:1496–503. doi: 10.1099/jmm.0.000179, PMID: 26445772

[55] Poolman JT Wacker M. Extraintestinal pathogenic *Escherichia coli*, a common human pathogen: challenges for vaccine development and Progress in the field. J Infect Dis. (2016) 213:6–13. doi: 10.1093/infdis/jiv429, PMID: 26333944 PMC4676548

[56] Cross JH Bradbury RS Fulford AJ Jallow AT Wegmuller R Prentice AM. Oral iron acutely elevates bacterial growth in human serum. Sci Rep. (2015) 5:16670. doi: 10.1038/srep16670, PMID: 26593732 PMC4655407

[57] Garber JJ Chung DC. Colonic polyps and polyposis syndromes In: Feldman M Friedman LS Brandt LJ, editors. Sleisenger and Fordtran's gastrointestinal and liver disease. 11th ed. Philadelphia, PA: Elsevier (2021)

[58] Diehl H. The iron reagents: Bathophenanthroline, bathophenanthroline-disulfonic acid, 2,4,6-tripyidyl-s-triazine [sic, and] phenyl-2-pyridyl ketoxime. 2nd ed. Columbus, Ohio: G.F. Smith Chemical Co. (1965).

[59] Babson AL Olson DR Palmieri T Ross AF Becker DM Mulqueen PJ. The Immulit assay tube: a new approach to heterogeneous ligand assay. Clin Chem. (1991) 37:1521–2. doi: 10.1093/clinchem/37.9.1521, PMID: 1893580

[60] Pereira DI Couto Irving SS Lomer MC Powell JJ. A rapid, simple questionnaire to assess gastrointestinal symptoms after oral ferrous sulphate supplementation. BMC Gastroenterol. (2014) 14:103. doi: 10.1186/1471-230X-14-103, PMID: 24899360 PMC4082414

[61] REDCap Software (2020). Available at: https://projectredcap.org/software/

[62] Clark MA Goheen MM Fulford A Prentice AM Elnagheeb MA Patel J. Host iron status and iron supplementation mediate susceptibility to erythrocytic stage *Plasmodium falciparum*. Nat Commun. (2014) 5:4446. doi: 10.1038/ncomms5446, PMID: 25059846 PMC4249681

[63] von Arnim U Wex T Ganzert C Schulz C Malfertheiner P. Fecal calprotectin: a marker for clinical differentiation of microscopic colitis and irritable bowel syndrome. Clin Exp Gastroenterol. (2016) 9:97–103. doi: 10.2147/CEG.S97701, PMID: 27147826 PMC4849404

[64] CRAN Package emmeans (1980). Available at: https://CRAN.R-project.org/package=emmeans

[65] Tolkien Z Stecher L Mander AP Pereira DI Powell JJ. Ferrous sulfate supplementation causes significant gastrointestinal side-effects in adults: a systematic review and meta-analysis. PLoS One. (2015) 10:e0117383. doi: 10.1371/journal.pone.0117383, PMID: 25700159 PMC4336293

