# Corrigendum: Safe and effective delivery of supplemental iron to healthy adults: a two-phase, randomized, double-blind trial – the safe iron study

**DOI:** 10.3389/fnut.2024.1376599

**Published:** 2024-02-27

**Authors:** Erin D. Lewis, Edwin F. Ortega, Maria Carlota Dao, Kathryn Barger, Joel B. Mason, John M. Leong, Marcia S. Osburne, Loranne Magoun, Felix J. Nepveux, Athar H. Chishti, Christopher Schwake, Anh Quynh, Cheryl H. Gilhooly, Gayle Petty, Weimin Guo, Gregory Matuszek, Dora Pereira, Manju Reddy, Jifan Wang, Dayong Wu, Simin N. Meydani, Gerald F. Combs

**Affiliations:** ^1^Jean Mayer USDA Human Nutrition Research Center on Aging, Tufts University, Boston, MA, United States; ^2^Department of Molecular Biology and Microbiology, Tufts University, Boston, MA, United States; ^3^Department of Developmental, Molecular and Chemical Biology, Tufts University, Boston, MA, United States; ^4^Department of Pathology, University of Cambridge, Cambridge, United Kingdom; ^5^Department of Food Science and Human Nutrition, Iowa State University, Ames, IA, United States

**Keywords:** iron, ferrous sulfate, malarial infectivity, bacterial proliferation, gut inflammation, IHAT, fungal iron

In the published article, there was an error. The error involved an inconsistency between our abstract and discussion regarding the observed effects of one treatment on some secondary outcomes, GI symptoms.

A correction has been made to **Abstract, subsection Results**.

This section previously stated:

“Supplementation with any form of iron did not affect any primary endpoint. In Phase I, the frequency of gastrointestinal symptoms associated with FS was unaffected by dosing with MNP or weekly administration; but participants taking IHAT more frequently reported abdominal pain (27%, *p* < 0.008) and nausea (4%, *p* = 0.009) than those taking FS, while those taking ASP more frequently reported nausea (8%, *p* = 0.009). Surprisingly, only 9% of participants taking IHAT at 120 mg Fe/day (Phase II) reported abdominal pain and no other group reported that symptom.”

The corrected section appears below:

“Supplementation with any form of iron did not affect any primary endpoint. Regarding secondary endpoints, in Phase I participants taking IHAT more frequently reported abdominal pain (27%, *p* = 0.008) than other iron forms; those taking the weekly FS dose more frequently reported nausea (20%, *P* = 0.009) than the other forms and modes of administration. In phase II, no such differences were observed.”

The authors apologize for this error and state that this does not change the scientific conclusions of the article in any way. The original article has been updated.

