# Supplementary material for: Safe and effective delivery of supplemental iron to healthy adults: a two-phase, randomized, double-blind trial – the safe iron study
Source: Front Nutr. 2023 Oct 11;10:1230061. doi: 10.3389/fnut.2023.1230061 (PMC10603204; doi:10.3389/fnut.2023.1230061)
Supplement: Supplementary file 1 [file Table_1.DOCX]

***Supplementary Material***

Safe and Effective Delivery of Supplemental Iron to Healthy Adults: A Two-Phase, Randomized, Double-Blind Trial – the Safe Iron Study

Erin D. Lewis, Edwin F. Ortega, Maria Carlota Dao, Kathryn Barger, Joel B. Mason, John M. Leong, Marcia S. Osburne, Loranne Magoun, Felix J. Nepveux V, Athar H. Chishti, Christopher Schwake, Anh Quynh, Cheryl H. Gilhooly, Gayle Petty, Weimin Guo, Gregory Matuszek; Dora Pereira, Manju Reddy, Jifan Wang, Dayong Wu, Simin N. Meydani^*^, and [Gerald F. Combs](mailto:Gerald.Combs@tufts.edu), Jr.

*** Correspondence:** Corresponding Author: Simin Nibkin Meydani simin.meydani@tufts.edu

| **Supplemental Table 1.** Phase I results: *Ex vivo* proliferation of bacteria in presence of subjects' plasma **2-HRS POST-PRANDIAL** | | | | | | | | |
| --- | --- | --- | --- | --- | --- | --- | --- | --- |
| Bacterium | Growth Parameter | Week | Treatment | | | | | |
|  |  |  | placebo (n=26) | FS daily 60 mg Fe/d (n=24) | IHAT 60 mg Fe/d (n=26) | ASP 60 mg Fe/d (n=25) | FS+MNP 60 mg Fe/d (n=24) | FS weekly 420 mg Fe  (n=25) |
|  |  |  | means [95% CI] ^1^ | | | | | |
| *Escherichia coli* | max OD index | 0 | 0.39 [0.34,0.44] | 0.47 [0.40,0.54] | 0.38 [0.32,0.45] | 0.37 [0.33,0.41] | 0.44 [0.37,0.52] | 0.68 [0.62,0.74] |
|  |  | 4 | 0.39 [0.34,0.45] | 0.39 [0.33,0.44] | 0.34 [0.30,0.38] | 0.35 [0.31,0.40] | 0.37 [0.31,0.42] | 0.62 [0.55,0.70] |
|  |  | Δ^2^ | 0.003 [-0.03,0.04] | -0.08 [-0.15,-0.01] | -0.04 [-0.10,0.01] | -0.01 [-0.03,0.01] | -0.07 [-0.13,-0.02] | -0.05 [-0.11,0.002] |
|  |  |  |  |  |  |  |  |  |
|  | peak growth rate index^3^ | 0 | 0.58 [0.56,0.60] | 0.59 [0.57,0.61] | 0.38 [0.32,0.45] | 0.57 [0.55,0.59] | 0.58 [0.56,0.60] | 0.65 [0.61,0.69] |
|  |  | 4 | 0.59 [0.56,0.62] | 0.58 [0.57,0.60] | 0.34 [0.30,0.38] | 0.57 [0.55,0.59] | 0.57 [0.55,0.60] | 0.61 [0.58,0.65] |
|  |  | Δ | 0.01 [-0.004,0.03] | -0.01 [-0.03,0.01] | -0.04 [-0.10,0.01] | 0.01 [-0.003,0.01] | -0.01 [-0.02,0.01] | -0.04 [-0.08,0.01] |
|  |  |  |  |  |  |  |  |  |
|  | time to peak growth, hr^4^ | 0 | 0 | 0 | 0 | 0 | 0.06 [-0.06,0.18] | 0.004 [-0.004,0.01] |
|  |  | 4 | 0 | 0 | 0.03 [-0.03,0.09] | 0 | 0.02 [-0.02,0.05] | 0.03 [-0.01,0.07] |
|  |  | Δ | 0 | 0 | 0.03 [-0.03,0.09] | 0 | -0.04 [-0.13,0.04] | 0.02 [-0.01,0.06] |
|  |  |  |  |  |  |  |  |  |
| *Acinobacter baumanii* | max OD index | 0 | 0.71 [0.69,0.74] | 0.76 [0.74,0.79] | 0.70 [0.65,0.75] | 0.68 [0.66,0.71] | 0.72 [0.66,0.78] | 0.83 [0.79,0.87] |
|  |  | 4 | 0.71 [0.69,0.74] | 0.72 [0.68,0.76] | 0.70 [0.66,0.73] | 0.70 [0.68,0.73] | 0.72 [0.68,0.76] | 0.81 [0.77,0.85] |
|  |  | Δ | 0.002 [-0.02,0.03] | -0.04 [-0.08,0.00] | -0.004 [-0.04,0.04] | 0.02 [-0.01,0.05] | 0.004 [-0.03,0.04] | -0.03 [-0.06,0.01] |
|  |  |  |  |  |  |  |  |  |
|  | peak growth rate index | 0 | 0.68 [0.65,0.70] | 0.68 [0.66,0.69] | 0.66 [0.64,0.68] | 0.661 [0.64,0.68] | 0.68 [0.65,0.70] | 0.70 [0.68,0.72] |
|  |  | 4 | 0.68 [0.65,0.70] | 0.67 [0.65,0.68] | 0.66 [0.64,0.67] | 0.662 [0.64,0.69] | 0.68 [0.65,0.70] | 0.69 [0.67,0.71] |
|  |  | Δ | 0.00 [-0.003,0.004] | -0.01 [-0.02,-0.002] | -0.001 [-0.01,0.004] | 0.002 [-0.003,0.01] | 0.00 [-0.01,0.01] | -0.01 [-0.02,0.003] |
|  |  |  |  |  |  |  |  |  |
|  | time to peak growth, hr | 0 | 8.23 [7.81,8.65] | 7.55 [6.86,8.24] | 7.86 [7.49,8.24] | 8.04 [7.58,8.49] | 7.85 [7.32,8.39] | 5.71 [4.64,6.78] |
|  |  | 4 | 8.33 [7.90,8.77] | 8.27 [7.92,8.62] | 8.44 [7.98,8.90] | 8.28 [7.84,8.73] | 7.94 [7.52,8.35] | 6.62 [5.69,7.55] |
|  |  | Δ | 0.10 [-0.22,0.43] | 0.72 [0.01,1.43] | 0.58 [0.18,0.97] | 0.25 [-0.16,0.65] | 0.08 [-0.23,0.40] | 0.92 [-0.15,1.98] |
|  |  |  |  |  |  |  |  |  |
| *Klebsiella pneumoniae* | max OD index | 0 | 0.23 [0.19,0.26] | 0.26 [0.21,0.32] | 0.24 [0.20,0.27] | 0.24 [0.21,0.26] | 0.26 [0.21,0.31] | 0.47 [0.38,0.55] |
|  |  | 4 | 0.23 [0.19,0.27] | 0.22 [0.19,0.26] | 0.22 [0.19,0.25] | 0.25 [0.22,0.28] | 0.24 [0.20,0.28] | 0.40 [0.32,0.48] |
|  |  | Δ | 0.01 [-0.02,0.03] | -0.04 [-0.09,0.01] | -0.02 [-0.05,0.01] | 0.01 [-0.01,0.03] | -0.01 [-0.03,0.01] | -0.07 [-0.16,0.03] |
|  |  |  |  |  |  |  |  |  |
|  | peak growth rate index | 0 | 0.47 [0.46,0.49] | 0.48 [0.46,0.50] | 0.46 [0.46,0.47] | 0.48 [0.47,0.49] | 0.48 [0.47,0.50] | 0.51 [0.49,0.54] |
|  |  | 4 | 0.48 [0.46,0.49] | 0.47 [0.46,0.48] | 0.47 [0.46,0.47] | 0.48 [0.47,0.49] | 0.49 [0.47,0.50] | 0.50 [0.48,0.52] |
|  |  | Δ | 0.003 [-0.003,0.01] | -0.01 [-0.03,0.01] | 0.01 [0.00,0.01] | -0.002 [-0.01,0.01] | 0.01 [-0.003,0.01] | -0.01 [-0.04,0.01] |
|  |  |  |  |  |  |  |  |  |
|  | time to peak growth, hr | 0 | 1.11 [0.40,1.82] | 1.85 [0.41,3.30] | 1.46 [0.52,2.39] | 1.07 [0.38,1.75] | 1.89 [0.43,3.34] | 2.35 [0.91,3.78] |
|  |  | 4 | 1.11 [0.50,1.73] | 1.14 [0.51,1.78] | 1.73 [0.47,3.00] | 1.35 [0.58,2.13] | 1.38 [0.14,2.61] | 2.34 [0.73,3.95] |
|  |  | Δ | 0.001 [-0.67,0.67] | -0.71 [-2.25,0.83] | 0.28 [-0.37,0.92] | 0.29 [-0.13,0.70] | -0.51 [-1.79,0.77] | -0.01 [-1.74,1.72] |
|  |  |  |  |  |  |  |  |  |
| *Staphylococcus aureus* | max OD index | 0 | 0.67 [0.61,0.74] | 0.74 [0.69,0.79] | 0.71 [0.64,0.78] | 0.68 [0.60,0.75] | 0.74 [0.67,0.81] | 0.78 [0.73,0.84] |
|  |  | 4 | 0.71 [0.65,0.76] | 0.72 [0.64,0.79] | 0.73 [0.67,0.79] | 0.70 [0.63,0.77] | 0.73 [0.66,0.81] | 0.77 [0.70,0.83] |
|  |  | Δ | 0.04 [-0.01,0.08] | -0.02 [-0.07,0.02] | 0.02 [-0.02,0.07] | 0.02 [-0.01,0.05] | -0.01 [-0.06,0.04] | -0.02 [-0.05,0.01] |
|  |  |  |  |  |  |  |  |  |
|  | peak growth rate index | 0 | 0.76 [0.72,0.80] | 0.80 [0.73,0.86] | 0.75 [0.70,0.80] | 0.77 [0.72,0.83] | 0.78 [0.71,0.84] | 0.78 [0.72,0.84] |
|  |  | 4 | 0.78 [0.73,0.82] | 0.81 [0.74,0.88] | 0.76 [0.71,0.82] | 0.79 [0.73,0.86] | 0.81 [0.73,0.88] | 0.79 [0.73,0.86] |
|  |  | Δ | 0.01 [-0.01,0.04] | 0.01 [-0.02,0.05] | 0.02 [-0.02,0.05] | 0.02 [-0.01,0.05] | 0.03 [-0.00,0.06] | 0.02 [-0.02,0.05] |
|  |  |  |  |  |  |  |  |  |
|  | time to peak growth, hr | 0 | 0.34 [0.04,0.64] | 0.09 [-0.10,0.28] | 0.12 [-0.02,0.26] | 0.08 [-0.04,0.20] | 0.22 [-0.05,0.48] | 0.10 [-0.11,0.30] |
|  |  | 4 | 0.27 [0.01,0.52] | 0.00 [0.00,0.00] | 0.04 [-0.04,0.12] | 0.05 [-0.05,0.16] | 0.18 [-0.18,0.55] | 0.23 [-0.14,0.60] |
|  |  | Δ | -0.07 [-0.28,0.14] | -0.09 [-0.28,0.10] | -0.08 [-0.24,0.09] | -0.03 [-0.19,0.13] | -0.03 [-0.22,0.16] | 0.13 [-0.06,0.32] |
|  |  |  |  |  |  |  |  |  |
| *Salmonella typinurium* | max OD index | 0 | 0.70 [0.67,0.73] | 0.76 [0.73,0.79] | 0.71 [0.66,0.77] | 0.71 [0.68,0.75] | 0.72 [0.67,0.78] | 0.80 [0.76,0.84] |
|  |  | 4 | 0.69 [0.65,0.73] | 0.72 [0.68,0.77] | 0.69 [0.64,0.74] | 0.71 [0.67,0.75] | 0.69 [0.63,0.74] | 0.76 [0.71,0.81] |
|  |  | Δ | -0.01 [-0.04,0.02] | -0.04 [-0.09,0.01] | -0.02 [-0.07,0.03] | -0.01 [-0.03,0.02] | -0.04 [-0.09,0.02] | -0.04 [-0.09,0.01] |
|  |  |  |  |  |  |  |  |  |
|  | peak growth rate index | 0 | 0.59 [0.58,0.60] | 0.62 [0.59,0.65] | 0.59 [0.57,0.61] | 0.61 [0.59,0.63] | 0.61 [0.59,0.63] | 0.67 [0.62,0.72] |
|  |  | 4 | 0.61 [0.59,0.63] | 0.66 [0.61,0.70] | 0.63 [0.58,0.67] | 0.61 [0.59,0.64] | 0.62 [0.60,0.65] | 0.64 [0.60,0.69] |
|  |  | Δ | 0.02 [-0.001,0.04] | 0.03 [-0.01,0.08] | 0.04 [0.003,0.07] | 0.002 [-0.02,0.02] | 0.02 [-0.01,0.04] | -0.03 [-0.09,0.03] |
|  |  |  |  |  |  |  |  |  |
|  | time to peak growth, hr | 0 | 2.66 [1.74,3.58] | 2.28 [1.32,3.23] | 2.70 [1.69,3.71] | 1.46 [0.81,2.10] | 2.42 [1.54,3.30] | 2.59 [1.55,3.63] |
|  |  | 4 | 2.05 [1.23,2.87] | 1.51 [0.75,2.26] | 1.96 [1.01,2.91] | 1.41 [0.74,2.08] | 2.46 [1.39,3.54] | 3.26 [2.19,4.33] |
|  |  | Δ | -0.61 [-1.31,0.10] | -0.77 [-1.66,0.12] | -0.74 [-1.50,0.03] | -0.05 [-0.85,0.76] | 0.05 [-0.83,0.92] | 0.67 [-0.66,2.01] |
| ^1^Arithmetic means and 95% confidence intervals.  ^2^Δ = wk4 - wk0, paired difference  ^3^Ratio of slope of the growth curves (log OD *v* time [hrs]) at the steepest point of the exponential growth phase to that of control. Change values (Δ) are the ratios of peak growth rate indices, wk4/wk0.  ^4^Cells with a zero indicate that all samples had an estimate of time to peak growth of 0 hrs. | | | | | | | | |
| ANOVA results: Pairwise comparisons (FS v placebo, IHAT v placebo, ASP v placebo, IHAT v FS, ASP v FS) and (FS daily v FS+MNP, FS daily v FS weekly) were estimated in a linear mixed-effects model adjusted for age, sex, and BMI for each growth parameter. P values were corrected using Tukey HSD method according to pairwise comparisons among each comparison set.  Three comparisons were significant: *Acinobacter baumanii* ASP v FS daily in max OD index (p=0.041) and peak growth rate index (p=0.015), and FS daily v placebo in peak growth rate (p=0.037). | | | | | | | | |

| **Supplemental Table 2**. Phase I results: noninferiority tests between intervention agents and FS daily for the primary study outcomes | | | | | | | | |
| --- | --- | --- | --- | --- | --- | --- | --- | --- |
|  | IHAT  60 mg Fe/d | | ASP  60 mg Fe/d | | FS+MNP  60 mg Fe/d | | FS weekly  420 mg Fe | |
|  | DID | P value | DID | P value | DID | P value | DID | P value |
| Parasitemia % ratio (24hr/0hr), log units | -0.024 (-Inf, 0.22) | 0.25 | 0.15 (-Inf, 0.39) | 0.71 | -0.12 (-Inf, 0.13) | 0.10 | -0.14 (-Inf, 0.14) | 0.099 |
| Fecal calprotectin, log(μg/g) fresh wt | -0.32 (-Inf, 0.14) | 0.038 | -0.085 (-Inf, 0.36) | 0.16 | -0.17 (-Inf, 0.27) | 0.095 | -0.011 (-Inf, 0.39) | 0.21 |
| *Escherichia coli*, peak growth rate index | -0.0085 (-Inf, 2.8e-05) | 0.049 | -0.0062 (-Inf, 0.0019) | 0.10 | -0.0025 (-Inf, 0.0058) | 0.30 | 0.0025 (-Inf, 0.015) | 0.63 |
| *Salmonella typhimurium*, peak growth rate index | -0.036 (-Inf, -0.0021) | 0.0045 | -0.044 (-Inf, -0.0099) | 0.0016 | -0.017 (-Inf, 0.017) | 0.042 | -0.025 (-Inf, 0.0084) | 0.016 |
| DID: Difference in difference, comparing each intervention agent to FS daily, expressed as (wk 4 – wk 0) or (wk 4/wk 0), as appropriate for each outcome, with 1-sided 95% confidence interval. Noninferiority tests are performed when a noninferiority margin (50% of the 4 week change) could be established from estimated mean differences between placebo and FS daily. Larger values in the outcome variables indicate a less desirable effect, thus the alternative hypothesis for each test was specified as H_A_: μ_(iron suppl)_-μ_(FS daily)_ ≤ margin), that is, the difference between each iron supplementation group and FS daily is less than or equal to the noninferiority margin. | | | | | | | | |
